# Machine learning analysis and risk prediction of weather-sensitive mortality related to cardiovascular disease during summer in Tokyo, Japan

**DOI:** 10.1038/s41598-023-44181-9

**Published:** 2023-10-09

**Authors:** Yukitaka Ohashi, Tomohiko Ihara, Kazutaka Oka, Yuya Takane, Yukihiro Kikegawa

**Affiliations:** 1 Faculty of Biosphere-Geosphere Science, Okayama University of Science, Kita-Ku, Okayama City, Okayama Japan; 2 Graduate School of Frontier Sciences, The University of Tokyo, Kashiwa City, Chiba Japan; 3 Center for Climate Change Adaptation, National Institute for Environmental Studies (NIES), Tsukuba City, Ibaraki Japan; 4 Environmental Management Research Institute, National Institute of Advanced Industrial Science and Technology (AIST), Tsukuba City, Ibaraki Japan; 5 School of Science and Engineering, Meisei University, Hino City, Tokyo Japan

**Keywords:** Environmental health, Environmental impact

## Abstract

Climate-sensitive diseases developing from heat or cold stress threaten human health. Therefore, the future health risk induced by climate change and the aging of society need to be assessed. We developed a prediction model for mortality due to cardiovascular diseases such as myocardial infarction and cerebral infarction, which are weather or climate sensitive, using machine learning (ML) techniques. We evaluated the daily mortality of ischaemic heart disease (IHD) and cerebrovascular disease (CEV) in Tokyo and Osaka City, Japan, during summer. The significance of delayed effects of daily maximum temperature and other weather elements on mortality was previously demonstrated using a distributed lag nonlinear model. We conducted ML by a LightGBM algorithm that included specified lag days, with several temperature- and air pressure-related elements, to assess the respective mortality risks for IHD and CEV, based on training and test data for summer 2010–2019. These models were used to evaluate the effect of climate change on the risk for IHD mortality in Tokyo by applying transfer learning (TL). ML with TL predicted that the daily IHD mortality risk in Tokyo would averagely increase by 29% and 35% at the 95th and 99th percentiles, respectively, using a high-level warming-climate scenario in 2045–2055, compared to the risk simulated using ML in 2009–2019.

## Introduction

Weather- or climate-sensitive diseases developing from heat or cold stress threaten human health worldwide^1–3^. Serious increases in temperature owing to global warming and urban heat islands threaten human health during the summer season^4–6^. Higher risks for cardiovascular, cerebrovascular, and respiratory diseases as well as heatstroke in summer are caused by country- or urban-scale increases in temperature^7–9^. A meta-analysis^10^ from worldwide research revealed that cardiovascular mortality in people aged 65 + years increased by 3.44% (95% confidence interval [CI] 3.10–3.78) for each 1 °C increase in temperature, and cerebrovascular mortality increased by 1.40% (95% CI 0.06–2.75).

An estimated 17.9 million people died from cardiovascular diseases in 2019, accounting for 32% of all global deaths^11^. The future health risk induced by climate change and the aging society in many countries must be urgently assessed to protect human health. Prediction of the mortality or morbidity of cardiovascular diseases is important for assessing the risk to vulnerable people and can been performed using machine learning (ML)^12–14^. ML algorithms have better performance than statistical models, such as the generalised linear model (GLM) and generalised additive model (GAM), in predictions of cardiovascular mortality^15^.

Japan’s super-aging society is unprecedented, and by 2050, it is estimated that the populations of people aged 65 + and 75 + years will represent 37.7% and 23.7%, respectively, of the total population^16^ (Fig. S1). In Japan, cardiac and cerebrovascular disease deaths, most of which occur among older people, accounted for 22.7% of all deaths in 2019, and were second to malignant neoplasm as the most frequent (27.3%) cause of death^17^. The Japanese government has reported that the number of patients hospitalised owing to cardiovascular diseases in 2035 will be twofold that in 2005, and the prevalences of cardiac and cerebrovascular diseases are estimated to increase 2.15- and 2.05-fold, respectively, by 2055^18^. ML techniques can be used to address summer heatstroke^19,20^, although no study has applied these techniques to the mortality or morbidity risk for cardiovascular diseases related to summer weather.

Quantitatively evaluating cardiovascular disease risk is also important to future society; hence, we sought to evaluate the future risk using an ML approach. In this study, we focused on cardiovascular diseases such as myocardial infarction and cerebral infarction, which are sensitive to weather or climate^21–23^ and predicted the mortality of these diseases in large Japanese cities using an ML technique and the data on weather parameters.

## Results

We evaluated ischaemic heart disease (IHD) and cerebrovascular disease (CEV) from all cardiovascular diseases (see “Methods”). The summer IHD and CEV mortalities in Tokyo’s 23 wards (hereafter, Tokyo) and Osaka City (hereafter, Osaka) were analysed for July–August of summer months during 2009–2019. The populations of Tokyo and Osaka in 2015 were approximately 9.3 million and 2.7 million, respectively.

**Figure 1:**
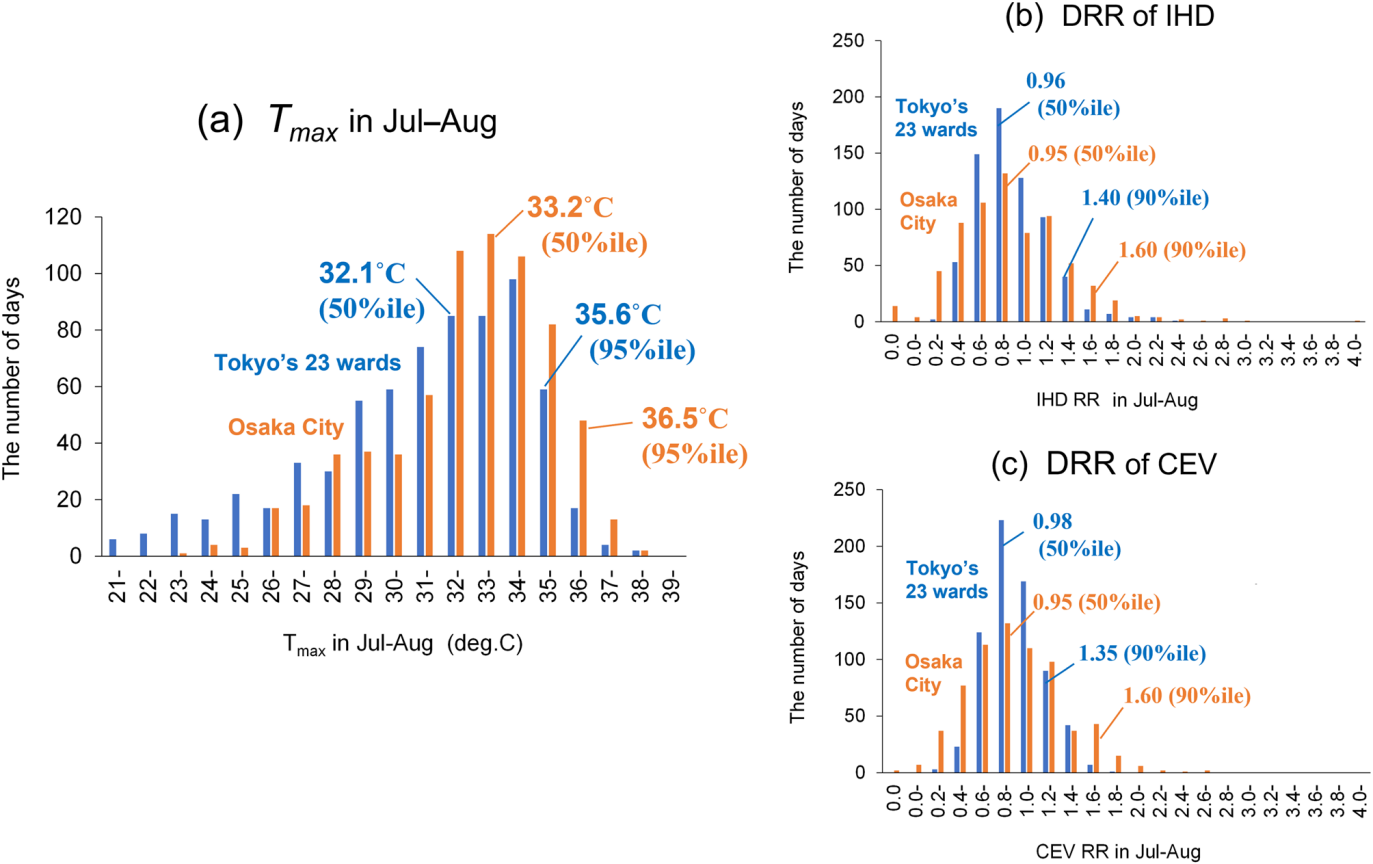
Frequency of (**a**) *T*_*max*_, (**b**) DRR of IHD, and (**c**) DRR of CEV during 2009–2019 in Tokyo’s 23 wards and Osaka City. *T*_*max*_ at the 50th and 95th percentiles and DRR at the 50th and 90th percentiles are shown in the respective graphs. *T*_*max*_ daily maximum temperature, *DRR* daily relative risk, *IHD* ischaemic heart disease, *CEV* cerebrovascular disease.

Figure 1 gives basic information on the daily maximum temperature (*T*_*max*_) and daily relative risk (DRR; normalised by yearly mean deaths in July–August) of IHD and CEV in July–August. The *T*_*max*_ in Tokyo was approximately 1°C lower than that in Osaka at both of the 50th and 95th percentiles (Fig. 1a). While the DRRs of IHD and CEV at the 50th percentile were almost identical in Tokyo and Osaka, those at the 90th percentile in Tokyo were 1.14- to 1.19-fold lower than in Osaka (Fig. 1b,c). For example, the mortality of IHD and CEV in people of ages 65 + years in Tokyo accounted for 85.5% and 88.0% of the total in 2009–2019, of which 76.5% and 83.3% were in people of ages 75 + years. Hence, we additionally focused on people aged 65 + and 75 + years, because the risk for heat-related morbidity or mortality from cardiovascular diseases is higher in older people^10^.

### Lag effect of weather exposure on mortality risk

**Figure 2:**
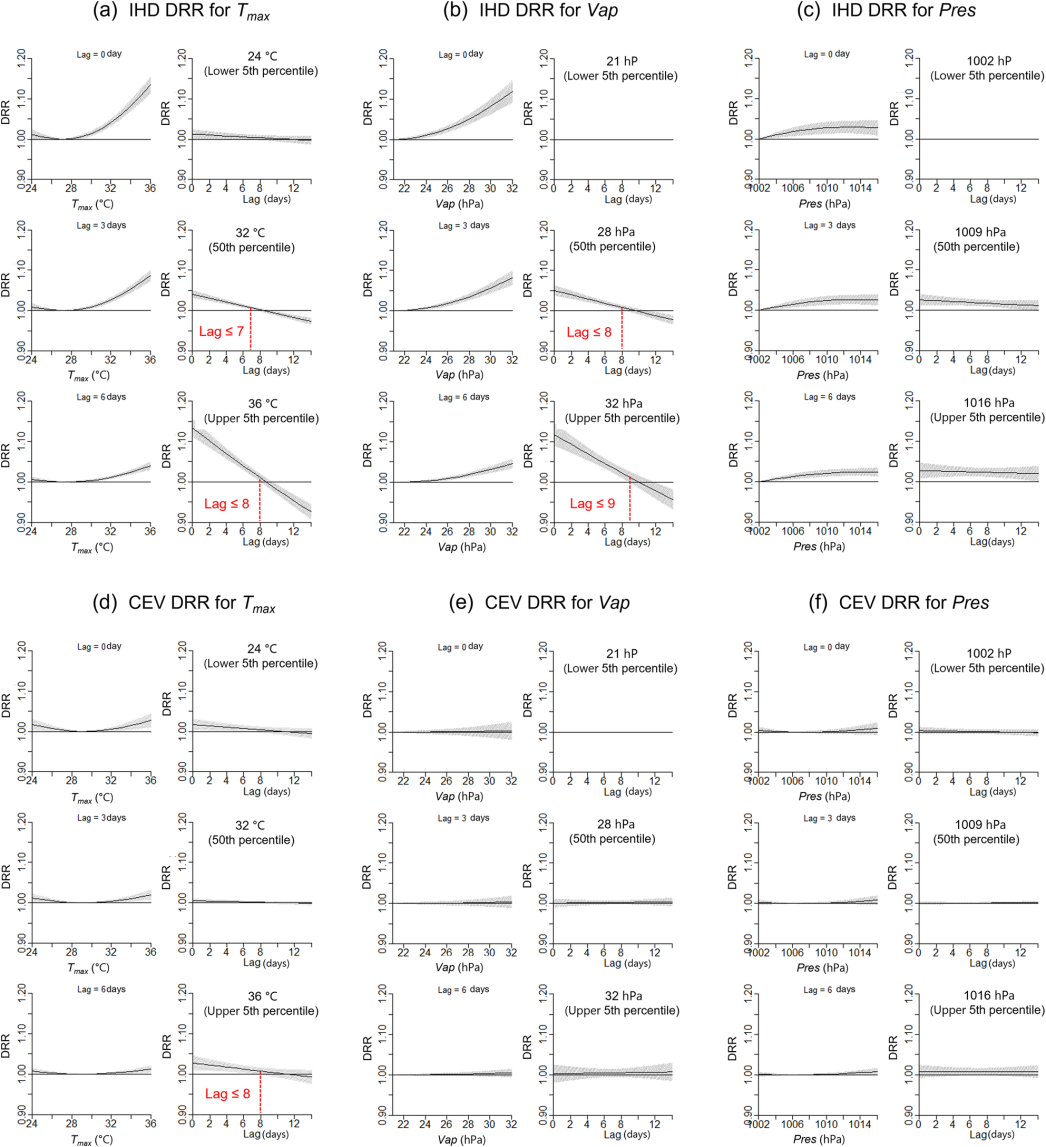
Results of lag analysis using the DLNM in Tokyo. (**a**–**c**) DRR of IHD and (**d**–**f**) DRR of CEV for (**a**,**d**) *T*_*max*_, (**b**,**e**) *Vap*, and (**c**,**f**) *Pres*. Each panel indicates (left) weather variables versus DRR at lags of 0, 3, 6 days and (right) lag days versus DRR at the lower 5th, 50th, and upper 5th (95th) percentiles of weather variables. *T*_*max*_ daily maximum temperature; *Vap*, daily mean water vapor pressure; *Pres*, daily mean air pressure; DLNM, distributed lag nonlinear model; DRR, daily relative risk; IHD, ischaemic heart disease; CEV, cerebrovascular disease.

The significance of delayed effects of daily weather conditions on mortality has been previously investigated using a distributed lag nonlinear model (DLNM)^24^ (see “Methods”). The results showed that the DRR of IHD increased rapidly with *T*_*max*_ and daily mean water vapor pressure (*Vap*); *T*_*max*_ and *Vap* exceeding 30 °C and 24 hPa, respectively, caused an exponential increase in IHD mortality risk in Tokyo (Fig. 2a,b; Fig. S2a–c for Osaka). In addition, the DRR remained higher with a higher *T*_*max*_ or *Vap* delayed for > 1 week. Although the DRR of the IHD response to daily mean air pressure (*Pres*) was less sensitive than that to *T*_*max*_ and *Vap* (Fig. 2c), the mortality risk persisted for > 10 days longer than those of *T*_*max*_ and *Vap*, with a higher *Pres*.

The response of the CEV DRR to weather exposure was significantly weaker than that of IHD (Fig. 2d–f). However, the overall risk for CEV, which was integrated for all lag effects, was present for weather elements (Fig. S3).

Table 1 summarises the set of lag days used for the subsequent ML, based on the DLNM results. Here, lag days were specified for people aged all, aged 65 + years, and aged 75 + years (Fig. S4–S7 from DLNM results). If the lag effect on the mortality risk with weather exposure was longer than the maximum of 14 days used in the DLNM analyses, it was assigned as 14 days in the ML features. The existence of lag days suggests that weather features on previous days should be incorporated into ML (e.g., *T*_*max*_ on the previous 1 day, 2 days, …, and 8 days for the DRR of IHD in Tokyo). Therefore, based on the data in Table 1, the *T*_*max*_, *Vap*, and *Pres* on previous days were added to the weather features used in ML implementation (Table 2).

**Table 1:** Lag days (numerals) of IHD and CEV mortality risks with weather exposure, determined by DLNM analyses. Representative results are shown for all ages, ages 65 + years, and ages 75 + years.

Daily weather elements	Tokyo’s 23 wards						Osaka City					
	All ages		Ages 65 +		Ages 75 +		All ages		Ages 65 +		Ages 75 +	
	IHD	CEV	IHD	CEV	IHD	CEV	IHD	CEV	IHD	CEV	IHD	CEV
*T*_*max*_	8	8	8	8	8	8	8	14	8	14	7	14
*Vap*	9	14	9	14	9	14	10	14	11	14	12	14
*Pres*	14	14	14	14	14	8	14	14	14	14	14	14

*T*_*max*_ daily maximum temperature, *Vap* daily mean water vapor pressure, *Pres* daily mean air pressure, *DLNM* distributed lag nonlinear model, *DRR* daily relative risk, *IHD* ischaemic heart disease, *CEV* cerebrovascular disease.

**Table 2:** Inputted initial features in ML and feature selection using BorutaSHAP. BorutaSHAP, Boruta SHapley Additive exPlanations.

	Initially inputted feature variables	Remarks
*T*_*max*_	*T*_*max*_, *T*_*max*_*Pre2*,…, *T*_*max*_Pre** *T*_*max*_*DiffPre1*,…,* T*_*max*_DiffPre** *AcT*_*max*_*30*	*T*_*max*_ : Daily maximum temperature (°C) Pre** : Previous ** day specified by DLNM (Table 1) DiffPre** : Difference from previous ** day specified by DLNM (Table 1) *AcT*_*max*_*30* : Accumulated high temperature (°C) defined by ${\sum }_{i}\left({T}_{max, i}-30.0\right)$ *i* : target date *Vap* : Daily mean vapor pressure (hPa) *Pres* : Daily mean air pressure (hPa) *Rain* : Daily accumulated rainfall (mm) *DOW* : Day of week
*Vap*	*Vap*, *VapPre2*,…,VapPre** *VapDiffPre1*,…, VapDiffPre**	
*Pres*	*Pres*, *PresPre2*,…, PresPre** *PresDiffPre1* , … , PresDiffPre**	
Others	*Rain* *DOW*	

### Mortality hindcast with weather features

A mortality hindcast for 2009–2019 was performed using ML techniques with Boruta SHapley Additive exPlanations (BorutaSHAP)^25^ for feature selection (see “Methods”). A gradient boosting algorithm (LightGBM)^26^ was adopted as the ML method in this study (see “Methods”). The inputted initial features are listed in Table 2. The temperature-related features include *T*_*max*_ on the day (*T*_*max*_), *T*_*max*_* n* days ago (*T*_*max*_*Pre n*), the difference from *n* days ago (*T*_*max*_*DiffPre n*), and accumulated high temperature (*AcT*_*max*_*30*) defined by:

1 $${AcT}_{max}30=\sum_{i}\left({T}_{max,i}-30.0\right)$$

Here, *i* represents the target date. Vapor- and air pressure-related features, daily rainfall, and the day of week were also used as initially inputted features (Table 2).

**Figure 3:**
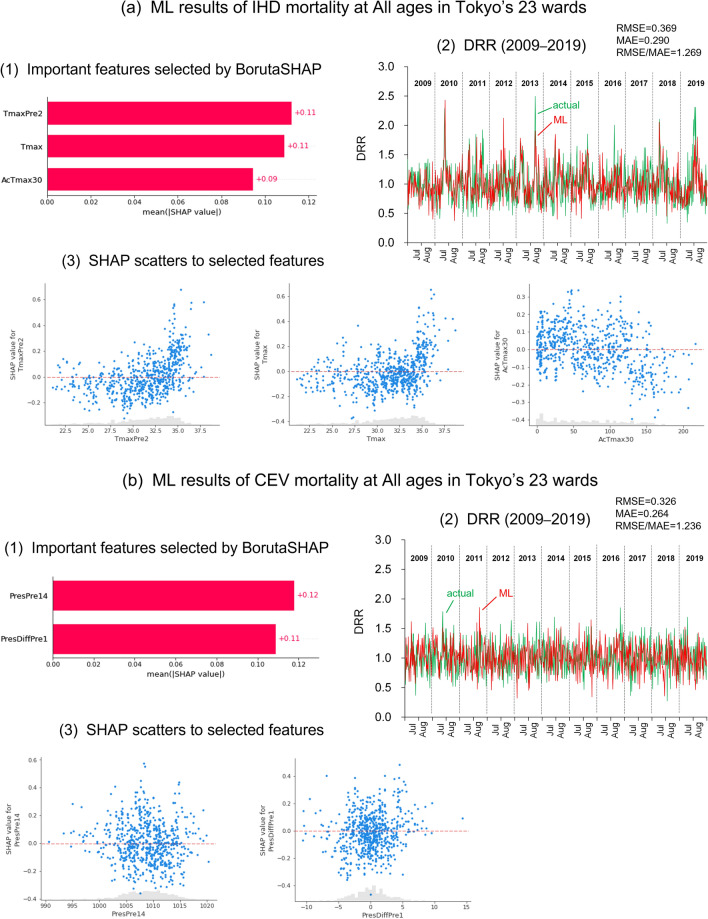
ML hindcast of (**a**) IHD and (**b**) CEV mortality during summer in Tokyo (2009–2019). (1) Important features selected using BorutaSHAP, (2) comparison of the simulated and actual DRR, and (3) relationships between SHAP values and the selected important features. ML, machine learning; BorutaSHAP, Boruta SHapley Additive exPlanations; *DRR,* daily relative risk; *IHD,* ischaemic heart disease; *CEV,* cerebrovascular disease; RMSE, root mean square error; MAE, mean absolute error.

Figure 3 shows the results of ML and the selected features for Tokyo IHD mortality at all ages (Fig. S8a,b for ages 65 + years and 75 + years). In the BorutaSHAP analysis, *T*_*max*_, *T*_*max*_*Pre2*, and *AcT*_*max*_*30* were selected from among the 37 features important for reproducing the DRR of IHD in Tokyo during the summer of each year (Fig. 3a-1). The model learning using these features related to temperature accurately reproduced the increases and decreases of the DRR in each year (Fig. 3a-2). Quantitative evaluation between the observed and simulated DRR yielded a root mean square error (RMSE) of 0.369, mean absolute error (MAE) of 0.290, and their ratio (RMSE/MAE) of 1.269.

The daily instance of the SHAP^26^ value can explain the quantitative attribution of a selected feature. The positive values of SHAP, which indicate an increased DRR, increased rapidly when *T*_*max*_*Pre2* or *T*_*max*_ exceeded approximately 34 °C (left and centre panels in Fig. 3a-3). However, an increase in *AcT*_*max*_*30* decreased the SHAP value (right panel in Fig. 3a-3), and the DRR of IHD was less likely to increase if *AcT*_*max*_*30* exceeded 130 °C. Meanwhile, ML implemented for Osaka selected only *T*_*max*_ as an important feature for DRR, and the RMSE and MAE between the actual and simulated DRR were larger than those of Tokyo (Fig. S8c–e).

Optimal features for the DRR of CEV in Tokyo were not temperature-related features but rather air pressure-related PresDiffPre1 and PresPre14 (Fig. 3b-1). Although the reproduced DRR of CEV (Fig. 3b-2) indicated an RMSE of 0.326, MAE of 0.264, and RMSE/MAE of 1.236, which were nearly equivalent to those of the aforementioned IHD, the responses of SHAP to the two pressure-related features were less sensitive than those of IHD (Fig. 3b-3). The simulated DRR for people aged 65 + years was also not related to the selected features (Fig. S9a,b) whereas the ML for DRR of people aged 75 + years failed to select important features via BorutaSHAP. Hence, it was difficult to perform ML prediction of CEV mortality in association with weather changes in Tokyo and Osaka.

### Future mortality risk

The sensitivity of IHD mortality risk to temperature-related features enabled estimation of changes in mortality risk caused by the hotter summers expected in the near future. Hence, the IHD mortality risk in Tokyo was evaluated with a sufficiently large population to avoid uncertainty. However, with a model trained using rare samples with a higher *T*_*max*_ and higher DRR in the present era, it is difficult to predict unknown or little-experienced future warming influences on DRR. Therefore, we used a resampling architecture (see “Methods”), padding the rare samples to balance their appearance prior to executing ML, and transfer learning (TL) architecture (see “Methods”) for extrapolation from the past training data, in future risk estimations.

**Figure 4:**
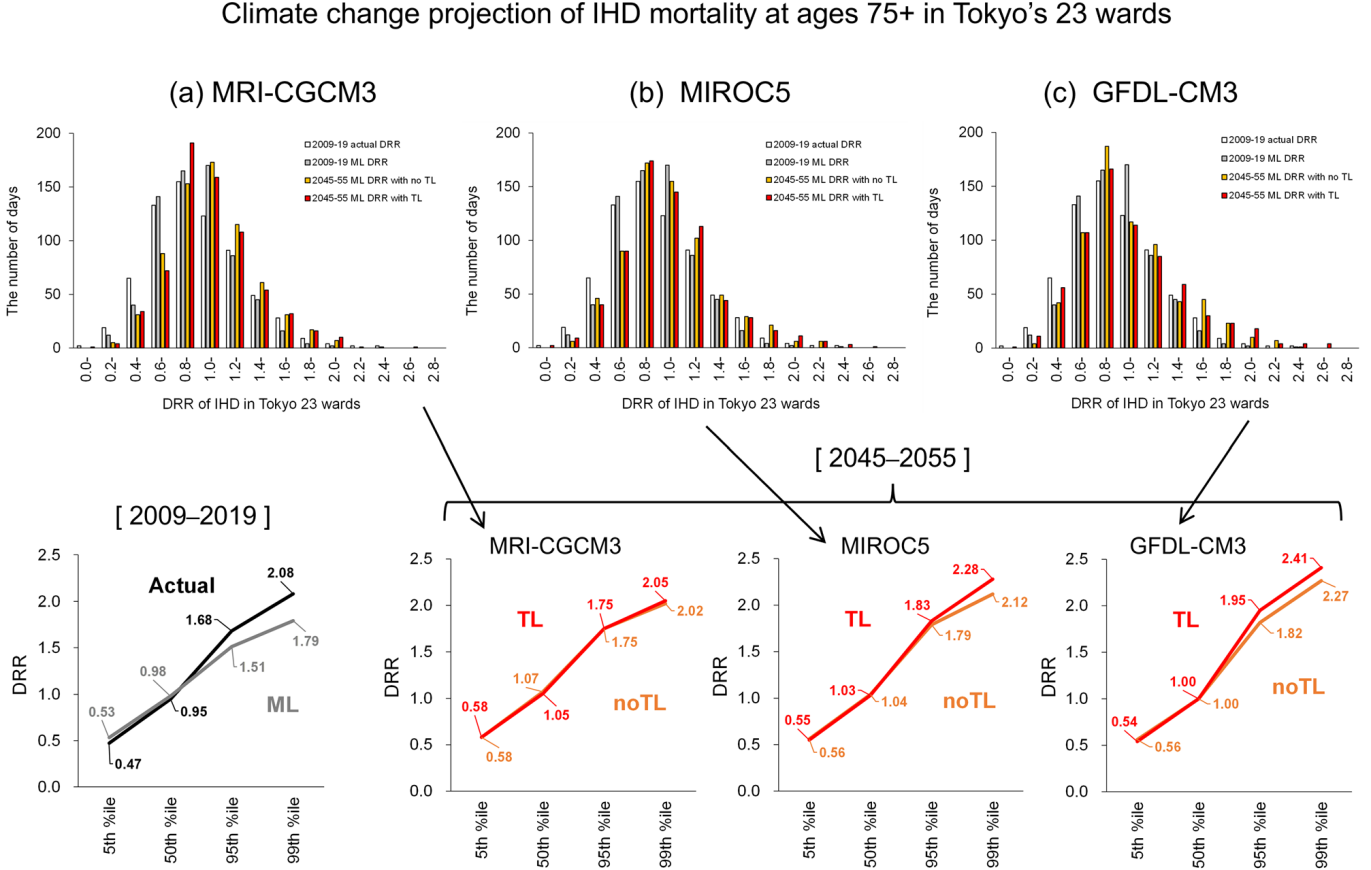
Frequency distributions (upper panels) and percentiles (lower panels) of the DRR of IHD for people aged 75 + years in Tokyo. Against actual and ML results in 2009–2019, the DRR change was estimated using climate projections of (**a**) MRI-CGCM3, (**b**) MIROC5, and (**c**) GFDL-CM3 under the RCP8.5 condition. “no TL” and “TL” indicate ML cases not incorporating TL and incorporating TL, respectively. *ML* machine learning, *DRR* daily relative risk, *IHD* ischaemic heart disease, *TL* transfer learning.

Figure 4 shows the effect of climate change over the next 20–30 years on Tokyo IHD mortality in people aged 75 + years (Fig. S10 for people aged 65 + years), which is expected to increase (Fig. S1). ML based on a model learned using the 2009–2019 dataset with the three important features (*T*_*max*_, *T*_*max*_*Pre2*, and *AcT*_*max*_*30*) was performed using future climate data for 2045–2055, predicted by the three global climate models: MRI-CGCM3 (Fig. 4a), MIROC5 (Fig. 4b), and GFDL-CM3 (Fig. 4c) under the RPC8.5 scenario from the NARO climate projection scenario dataset^27^ (see “Methods”). The temperatures predicted by the three models are known as the lowest (MRI-CGCM3), middle (MIROC5), and highest (GFDL-CM3) increasing tendencies from the present period (Fig. S10). Comparison with the DRR in 2009–2019 (the lower panels in Fig. 4) showed that each percentile of the estimated future DRR (approximately 30 years later) was higher than the present percentile for all of the climate models. The smallest increase in the future era was estimated to be 1.16-fold (1.04-fold) at the 95th percentile, corresponding to the upper 5% of overall days compared to the ML-simulated (actual) DRR at the present era, and 1.13-fold (0.97-fold) at the 99th percentile corresponding to the upper 1% of overall days. On the other hand, the largest increase was anticipated to be 1.29-fold (1.16-fold) at the 95th percentile and 1.35-fold (1.16-fold) at the 99th percentile compared to the ML-simulated (actual) DRR in the present era.

The effectiveness of TL using data of a hotter region (i.e., Osaka) in future warming projections for Tokyo is also indicated as “no TL” and “TL” in Fig. 4. Their comparison clarified that TL cases increased the DRR values relative to no TL cases in warmer climate models. This suggests that learning using high temperatures is needed for ML to perform well conditions of little experience with a future warmer climate in a target region (i.e., Tokyo). Incorporation of TL increased DRR by 2.2% and 7.5% at the 95th and 99th percentiles, respectively, compared to without TL, for the middle-level warming climate of MIROC5, and those increased DRR by 7.1% and 6.2% for the high-level warming climate of GFDL-CM3 (the lower panels in Fig. 4). Finally, ML incorporating TL showed that the daily IHD mortality risk in Tokyo on average increased by 29% and 35% at the 95th and 99th percentiles using the high-level warming climate scenario in 2045–2055, compared to the risk simulated using ML in 2009–2019.

## Discussion

Pre-analyses using the DLNM suggested a requirement of lag-related weather elements for daily mortality when selecting features for inclusion in ML. Lag days of approximately 1 week for heat exposure (*T*_*max*_ in this study) in IHD mortality (Fig. 2a–c) are supported by the results of a meta-analysis^28^ of research conducted in several countries. This 1-week delay of the mortality risk for IHD in summer is longer than that for heatstroke (0–2 days)^29^. The increase in cardiovascular disease mortality at higher temperatures is attributed to dehydration-induced increases in the viscosity of plasma, serum cholesterol levels, and red blood cell and platelet counts^21,30^. In addition, the increase in core body temperature caused by an exaggerated thermoregulatory response can lead to the development of acute cardiovascular diseases^30^.

Although CEV mortality is less sensitive to weather elements (Fig. 2d–f), the lag effect of *T*_*max*_ and the longer-delayed effect of *Pres* were found to have weak responses in the DRR. In particular, pressure-related features tended to be selected as important features for the ML of CEV, instead of temperature-related features (Fig. 3b). A nonsignificant effect of high temperature on CEV mortality has been reported in several studies^31^. Although changes in pressure are related to CEV diseases, such as subarachnoid haemorrhage^32^, associations with air pressure in summer have not been epidemiologically confirmed. These characteristics of CEV hamper reproduction of the DRR with weather features using ML.

An additional temperature effect of *AcT*_*max*_*30* is likely needed to reproduce the DRR of IHD in Tokyo. Indeed, peaks in the DRR in 2018 and 2019 were reproduced by the inclusion of *AcT*_*max*_*30* when executing ML (Fig. S11). As suggested by the SHAP response to *AcT*_*max*_*30*, an increase in *AcT*_*max*_*30* reduced the DRR of IHD (Fig. 3a-3), implying a kind of heat acclimatisation. Excessive heat loads to the human body have adverse effects on cardiovascular function (e.g., thermoregulatory disruption and haemoconcentration)^33^ whereas heat acclimatisation of the human body produces cardiovascular adaptation (improving physiological responses to heat)^34^, even with long-term heat exposure over several months^35^.

In this study, evaluations of future DRR possibilities using climate projection data were challenged for the IHD mortality risk in Tokyo. However, the influence of future further aging of the population on ML implementation was not considered. The DRR values defined in this study represent the relative mortality risks during summer of 1 year, to avoid the influence of year-to-year changes caused by, for example, natural progression of population aging and medical advances that extend longevity. However, the future DRR calculated for people aged 75 + years (Fig. 4) was probably underestimated in comparison with the actual DRR because of the growth of the population older people aged 85 + years.

TL has been applied to predict future change in various disciplines, such as Earth sciences^36–38^. In this study, we evaluated the change in mortality risk under future climate conditions, incorporating TL and imbalanced learning (or resampling)^39,40^ architectures. A similar method has been used to forecast extreme heatwaves^41^. Because the characteristics that related *T*_*max*_ to DRR in Osaka City (“source or supporting data” in TL) except for population size were similar to those in Tokyo’s 23 wards (“target data” in TL) (Fig. 1), ML implementation with TL was effective for higher-level climate warming (predicted by MIROC5 and GFDL-CM3) in Tokyo.

Because evaluation of the future mortality from ML and applying TL is breakthrough challenging, it is difficult to ensure the accuracy of the predicted mortality risk. However, use of the actual relationships between mortality risk and weather conditions in Osaka City, which are not currently experienced in Tokyo, can interpolatively predict a potential future mortality risk in Tokyo. TL was conducted using artificial data over-sampled around the upper 10% of the DRR in Tokyo, which was rare from 2009 to 2019. This resampling technique increased the DRR at the maximum frequency of appearance by 1.3-fold. Future deaths owing to IHD in Tokyo have not been officially analysed, whereas it is estimated that the number of inpatients with cardiovascular diseases will increase 1.3-fold by 2050 compared to 2015^18^. Hence, over-sampling at the upper 10% of DRR should be used in future investigations.

## Methods

### Daily weather data

**Figure 5:**
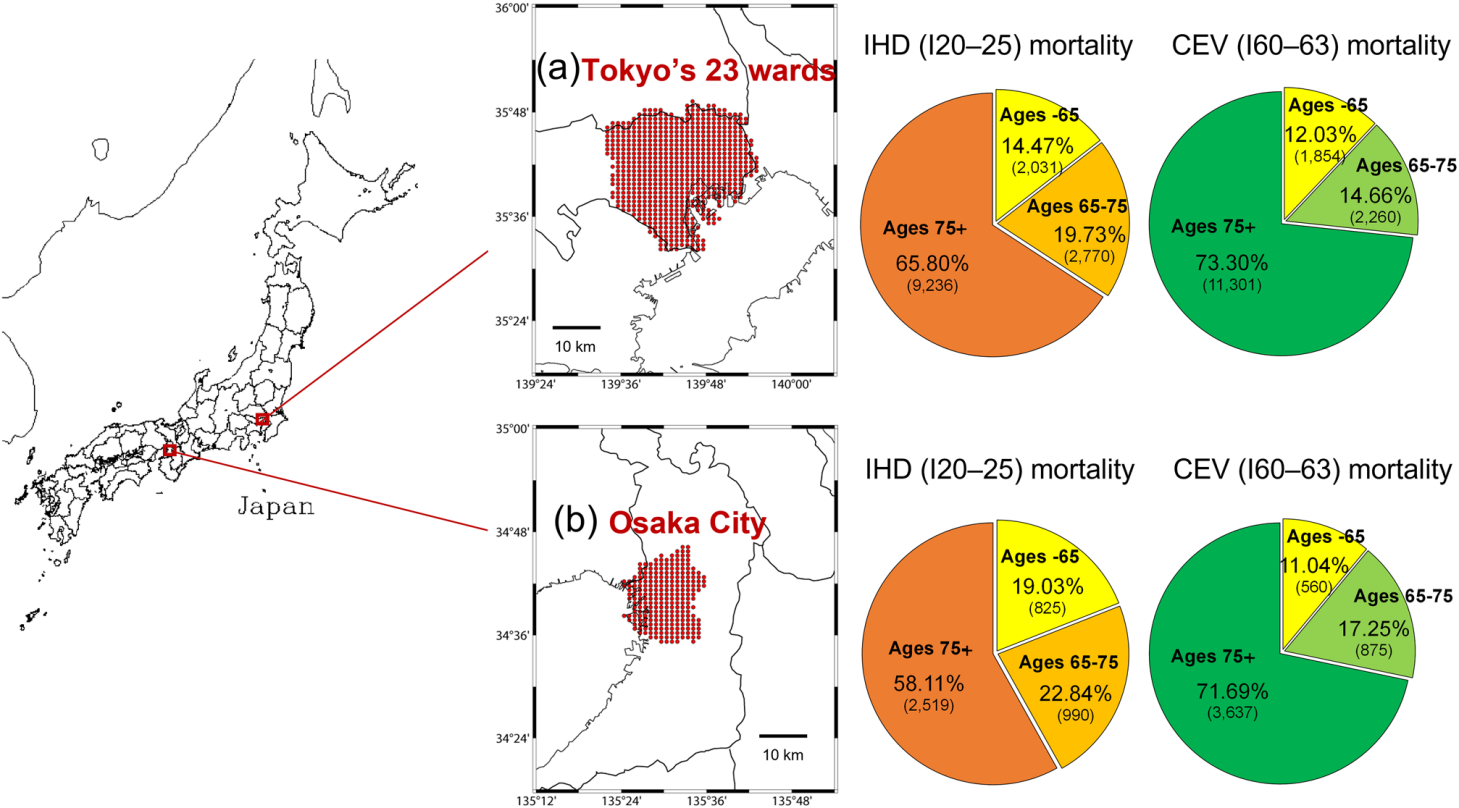
Weather data grids (red dots in maps at left) and summer ischaemic heart disease (IHD) and cerebrovascular disease (CEV) mortality rates according to age group (pie charts at right) analysed in (**a**) Tokyo and (**b**) Osaka. Gridded weather data at 1 km resolution from the Agro-Meteorological Grid Square Data provided by the NARO were used. Mortality data were extracted for July–August from 2009 to 2019. The Generic Mapping Tools (GMT) graphic system (version 6.4.0; https://www.generic-mapping-tools.org/team.html) was used to draw the maps.

Agro-Meteorological Grid Square Data (https://amu.rd.naro.go.jp/) provided by the National Agriculture and Food Research Organization (NARO)^42^ were used to assess daily weather conditions in Tokyo and Osaka. These data were developed by 1 km spatial interpolation of meteorological elements (e.g., temperature, wind speed, rainfall, and solar radiation) measured nationwide in Japan at observation stations of the Japan Meteorological Agency (JMA). Temperature-related data were corrected for grid altitude. In this study, *T*_*max*_ (°C) and *Rain* (mm) were extracted and averaged for grids corresponding to Tokyo’s 23 wards (986 grids) and Osaka City (422 grids), as shown in Fig. 5. *Pres* and *Vap* data were from the JMA observation station (https://www.jma.go.jp) located in the centre of Tokyo and Osaka, because the abovementioned Agro-Meteorological Grid Square Data do not include those elements.

### Daily mortality data

Statistical surveillance information regarding the number of daily deaths (https://www.e-stat.go.jp/en) published by the Ministry of Health, Labour and Welfare (MHLW) of the Japanese government were used in this study. Information such as the cause of death, age, and sex was included in the data. The International Statistical Classification of Diseases and Related Health Problems 10th Revision (ICD-10) was used to classify causes of death. We analysed the following I20–25 and I60–63 codes corresponding to IHD and CEV, respectively: I20, angina pectoris; I21, acute myocardial infarction; I22, subsequent myocardial infarction; I23, certain current complications following acute myocardial infarction; I24, other acute ischaemic heart diseases; I25, chronic ischaemic heart disease; I60, subarachnoid haemorrhage (including sequelae, I69.0); I61, intracerebral haemorrhage (including sequelae, I69.1); I62, other nontraumatic intracranial haemorrhage (including sequelae, I69.2); and I63, cerebral infarction (including sequelae, I69.3). The mortality from IHD and CEV was approximately 60–70% in people aged 75 + years (Fig. 5).

### Lag analysis and machine learning

**Figure 6:**
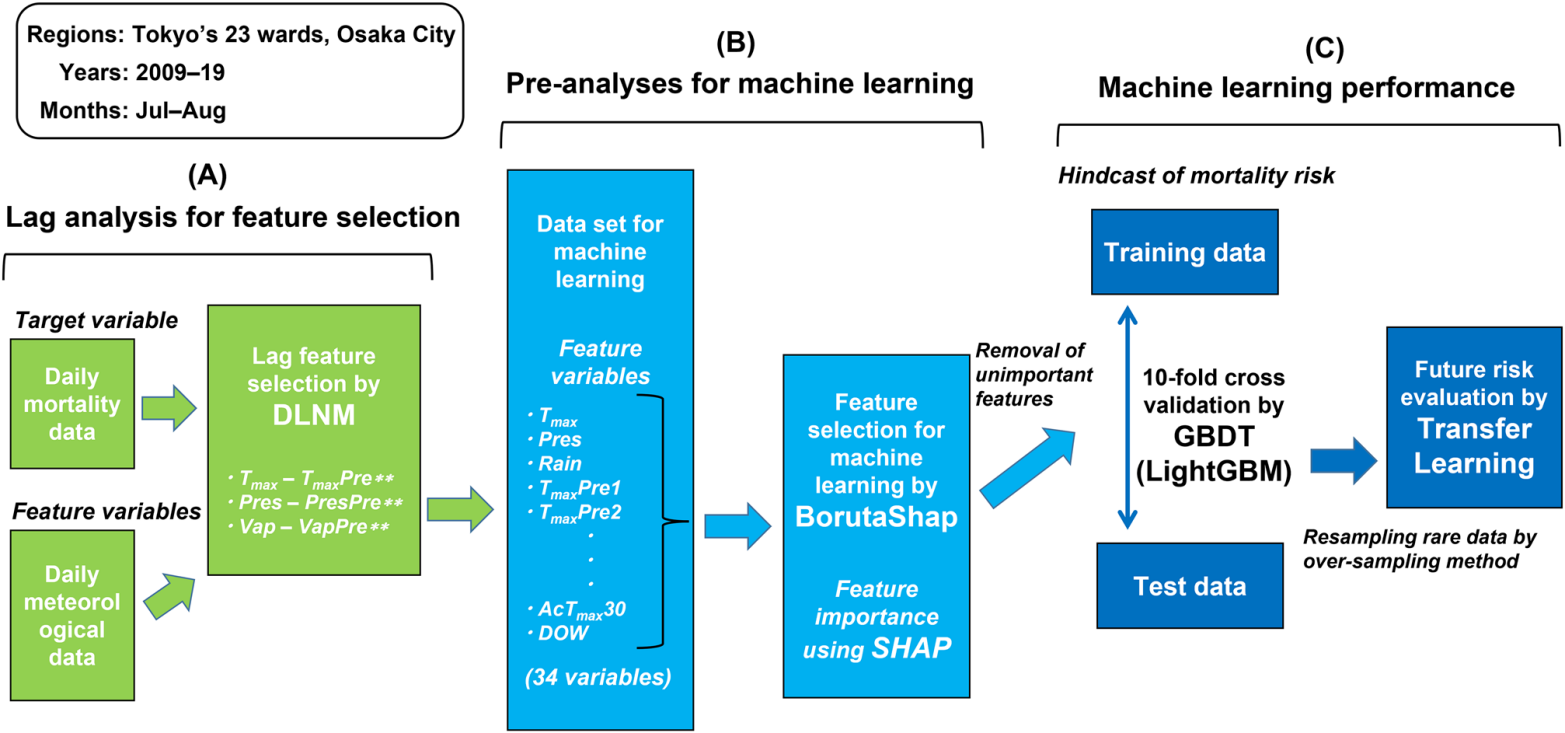
Flow of analysis in this study. We performed “lag analysis for feature selection”, “pre-analyses for machine learning”, and “machine learning performance”. DLNM, distributed lag nonlinear model; SHAP, SHapley Additive exPlanations; GBDT, gradient boosting decision tree.

Figure 6 depicts the flow of analysis in this study. We conducted: (A) a lag analysis for feature selection related to IHD or CEV mortality, (B) pre-analyses for ML, and (C) mortality hindcast and future risk evaluation using ML.

### Lag analysis

The DLNM^24^ was used to reveal a delayed weather effect on IHD and CEV mortality in the part (A) of Fig. 6. This model has been used in public health studies^43^ and is defined using the following model equation:

2 $$\mathrm{log}\left({ND}_{t}\right)=\mathrm{intercept}+{cb}_{t,l}+\mathrm{ns}\left(date, df\times year\right)$$

Here, *ND*_*t*_ represents the expected number of deaths at day *t* in which the error function was assumed to follow a quasi-Poisson distribution. *cb*_*t*,*l*_ is the cross-basis matrix for a weather variable (*T*_*max*_, *Vap*, or *Pres*) with *t* and lag days *l*, which is produced by the DLNM fitting the nonlinear and lag effects. ns means a natural spline function, which was examined for the date with the degree of freedom per year (*df* = 2) and *year* = 11. This term works to adjust long-term trends. However, the influence of day of the week on mortality was negligible according to sensitivity experiments including or excluding the term in Eq. (2). The exposure–response curves were modelled by a natural cubic function with 2 degrees of freedom for variables and lag days. Those knots were placed at equally-spaced values in the temperature range and at equal intervals on a logarithmic scale for lag days by default^24^.

The maximum number of lag days was assigned as 14 days (2 weeks), and two degrees of freedom were used for the weather variable and lag effect. Moghadamnia et al.^28^ revealed that temperature lag affected the risk for cardiovascular mortality year-round with the greatest risk at 14 lag days, by their systematic review and meta-analysis worldwide. Because heat-related mortality indicates shorter lag effects than cold-related mortality, we set 14 days as the maximum lag days. In addition, we assumed identical maximum lag effects of weather parameters other than temperature because lag effects on mortality risk have not been revealed.

The DLNM was conducted for one of the three weather variables (i.e., *T*_*max*_, *Vap*, or *Pres*) without incorporating the other two variables as confounders, because the specified lag days for selecting ML features were unaffected even if confounders were incorporated to implementations of DLNM.

### Feature selection

BorutaSHAP^25^, which combines the Boruta feature selection algorithm with SHAP, was also used in part (B) of Fig. 6. The Boruta feature selection is a wrapper method for detecting important features in ML^44,45^: Shuffled duplicates (shadow features as noise) of all features are added as unpredictability to the original feature dataset (e.g., *T*_*max*_, *T*_*max*_*Pre*2, …, *Pres*, *PresPre*2, …); next, feature importance based on Z-scores in the enlarged dataset (i.e., original features + shadow features) is used to train a decision tree-based algorithm (Gradient Boosting Decision Trees in this study). Each training cycle is analysed for a higher priority feature than the most important shadow feature, and elements considered highly irrelevant are deleted.

BorutaSHAP provides flexibility in model selection and allows visualisation of the selected features by applying the SHAP^25^. The SHAP, i.e., “Shapley value,” has been originally developed to estimate the contribution of an individual player in a collaborative team and ensure fair allocation according to their contribution^46,47^. Features (daily weather elements in this study) contribute to the model’s output or prediction with a different magnitude (importance) and sign (positive or negative), which is accounted for by the Shapley values^48^.

### Machine learning (ML)

A gradient boosting algorithm was adopted as the ML used in part (C) of Fig. 6; this is an ensemble learning technique to improve the performance of ML^49,50^. Ensemble learning includes multiple models termed “weak learners” (generally decision trees); their outputs are combined for prediction or classification problems^25^. Boosting learners learn in a sequential manner to correct errors from the previous learner and create a robust model to reduce model bias. Therefore, a gradient boosting ML increases accuracy more than other ML algorithms, such as random forest^49,51^. In this study, the LightGBM^26^ was adopted as a gradient boosting ML, which significantly outperforms other gradient boosting algorithms in terms of computational speed and memory consumption^52^.

The 2009–2019 dataset was divided into 10 groups and iteratively evaluated using a *k*-fold cross-validation method^53^ (i.e., *k* = 10), which used 90% of the data as training data and the remaining 10% as testing data. In searching the best hyperparameters in the ML model, “the number of leaves” parameter required for leaf-wise tree growth, which is adopted in LightGBM, was optimised from values of 10–100 for ML.

### Evaluation to future climate change

#### Transfer learning (TL)

A TL architecture^54–56^ was applied to evaluate future mortality in this study. Because the present era (2009–2019) data did not include many days with higher temperature which could happen frequently in the future climate, the future DRR evaluated by ML may be biased toward the present average temperatures. Therefore, to resample the present (2009–2019) data to the higher frequency of high-temperature appearance days in the future, the Synthetic Minority Over-Sampling Technique for Regression with Gaussian Noise (SMOGN)^57^ was implemented as a pre-processor for TL. Regression analysis often targets an accurate prediction of rarely occurring extreme values of an objective variable, which is assigned continuous values. To increase the frequency of rare instances (days with extremely high temperatures in this study), imaginary data were generated by applying Gaussian noise to the rare samples. Resamples using the SMOGN were adjusted to be over-sampled around the upper 10% of DRR in Tokyo, which is rare in the present era but could be more frequent in the future.

TL was applied to explore future possibilities for DRR of IHD in Tokyo, which can improve the prediction accuracy of a task for a target domain (Tokyo data) in conjunction with information obtained from a task for a source domain (Osaka data). This situation corresponds to a simple “homogeneous transfer” with transformation of the source domain to the target domain. If there is an available dataset drawn from a domain related to but not exactly matching a target domain of interest, homogeneous TL can be used to build a predictive model for the target domain as long as the input feature space is the same^55^. A feature-based algorithm of the Feature Augmentation Method^58^ was adopted as TL to evaluate changes in risk. The augmented source data contain common and source-specific domains, whereas the augmented target data contain common and target-specific domains. Hence, the feature dimension is augmented threefold ($$\chi \to \overset{\lower0.5em\hbox{$\smash{\scriptscriptstyle\smile}$}}{\chi }$$ $$={\mathbb{R}}^{3F}$$; χ denotes a feature domain, $$\overset{\lower0.5em\hbox{$\smash{\scriptscriptstyle\smile}$}}{\chi }$$ an augmented feature domain, and $${\mathbb{R}}^{3F}$$ a three-dimensional real space). Next, it is defined as Φ_*s*_ and Φ_*t*_ as mappings of the source and target data, respectively, for $$\chi \to \overset{\lower0.5em\hbox{$\smash{\scriptscriptstyle\smile}$}}{\chi }$$:

3 $${\Phi }_{s}\left({\varvec{x}}\right)=\langle {\varvec{x}},{\varvec{x}},\varvec{0}\rangle\, \mathrm{and}\,{\Phi }_{t}\left({\varvec{x}}\right)=\langle {\varvec{x}},\varvec{0},{\varvec{x}}\rangle$$

Here, **0** is the zero vector and ***x*** is the feature vector of the source or target domain. Finally, supervised learning was implemented by assigning the dataset of Tokyo as Φ_*t*_ and that of Osaka as Φ_*s*_.

#### Climate change projection

The Regional Climate Projection Scenario Dataset^27^ (https://amu.rd.naro.go.jp/) provided by the NARO was used to evaluate the effect of future climate change on the DRR of IHD in Tokyo. The output results, simulated using several global climate models, were statistically downscaled to the Japanese regional model with 1 km spatial resolution. A Gaussian-type scaling approach^59^ was adopted as a statistical downscaling method to improve the reproducibility of daily and annual variation. Based on the relationship between the standard deviations (e.g., temperature, wind speed, rainfall, and solar radiation) of the global climate model and observations for a past reference period, means and standard deviations were corrected such that the climate change signal would not be enhanced^60^.

From published output results of several models, the MRI-CGCM3 (Japan; Meteorological Research Institute), MIROC5 (Japan; The University of Tokyo, National Institute for Environmental Studies, and Japan Agency for Marine–Earth Science and Technology), and GFDL-CM3 (USA; NOAA Geophysical Fluid Dynamics Laboratory) models, which were used in the Coupled Model Intercomparison Project phase 5 (CMIP5)^61^, were chosen because the simulated temperature bias included low, middle, and high levels, respectively, of the NARO climate projection scenario dataset^62^ (cf. Fig. S12). In addition, these model projections included the two scenarios RCP2.6 (low-emissions scenario via stringent mitigation) and RCP8.5 (high-emissions scenario without any mitigation) of the greenhouse gas emissions “pathway”^63^. In this study, we used the projection result of RCP8.5 as the worst-case climate scenario to evaluate the future IHD risk.

### Supplementary Information

Supplementary Information.

## Data Availability

The data that support the findings of this study are available from the NARO portal site of official statistics published (gridded weather and climate scenario data; https://amu.rd.naro.go.jp/) and MHLW (death data; https://www.e-stat.go.jp/en). These data are of restricted availability, and we used them with permission for this study. Therefore, data are available from the corresponding author upon reasonable request and with the permission of the NARO and the MHLW.
