# Supplementary material for: Machine learning analysis and risk prediction of weather-sensitive mortality related to cardiovascular disease during summer in Tokyo, Japan
Source: Sci Rep. 2023 Oct 9;13:17020. doi: 10.1038/s41598-023-44181-9 (PMC10562479; doi:10.1038/s41598-023-44181-9)
Supplement: Supplementary file 1 — Supplementary Information. [file 41598_2023_44181_MOESM1_ESM.pdf]

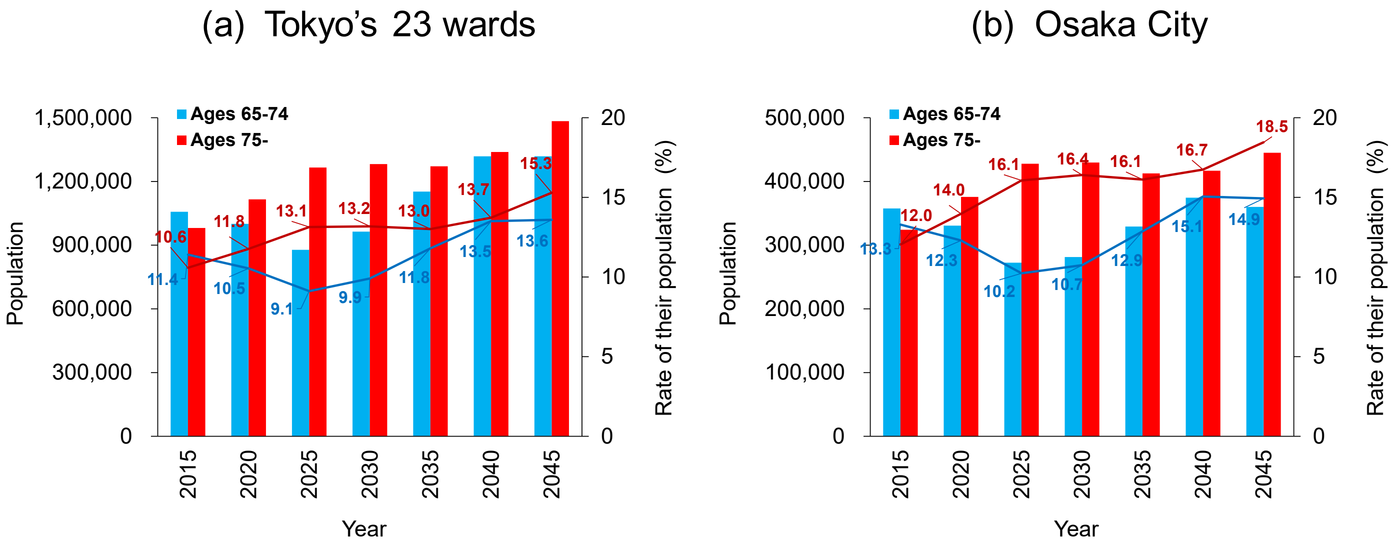

Supplementary Figure S1. Populations aged 65–74 and 75+ years and the rate of their populations to the all in 2015–2045 in (a) Tokyo's 23 wards and (b) Osaka City. The data were cited from the National Institute of Population and Social Security Research (https://www.ipss.go.jp/pp-shicyoson/j/shicyoson18/3kekka/Municipalities.asp).

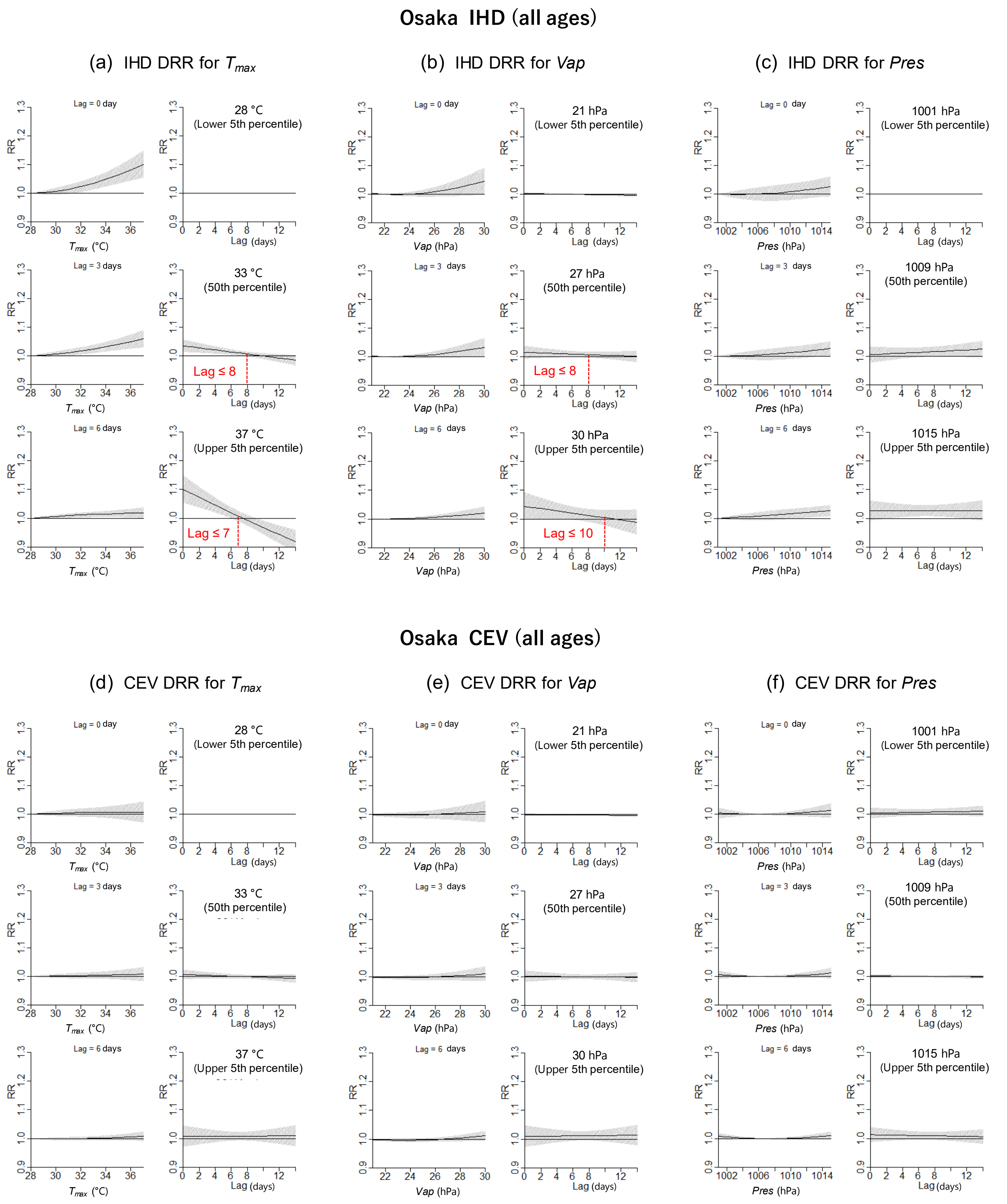

Supplementary Figure S2. Results of lag analysis using the DLNM in Osaka City. (a)–(c) DRR of IHD and (d)–(f) DRR of CEV for (a)(d) T_{max}, (b)(e) *Vap*, and (c)(f) *Pres*. Each panel indicates (left side in the panel) weather variables versus DRR at lags of 0, 3, 6 days and (right side in the panel) lag days versus DRR at the lower 5th, 50th, and upper 5th (95th) percentiles of weather variables. T_{max}, daily maximum temperature; *Vap*, daily mean water vapor pressure; *Pres*, daily mean air pressure; DLMN, distributed lag nonlinear model; DRR, daily relative risk; IHD, ischaemic heart disease; CEV, cerebrovascular disease.

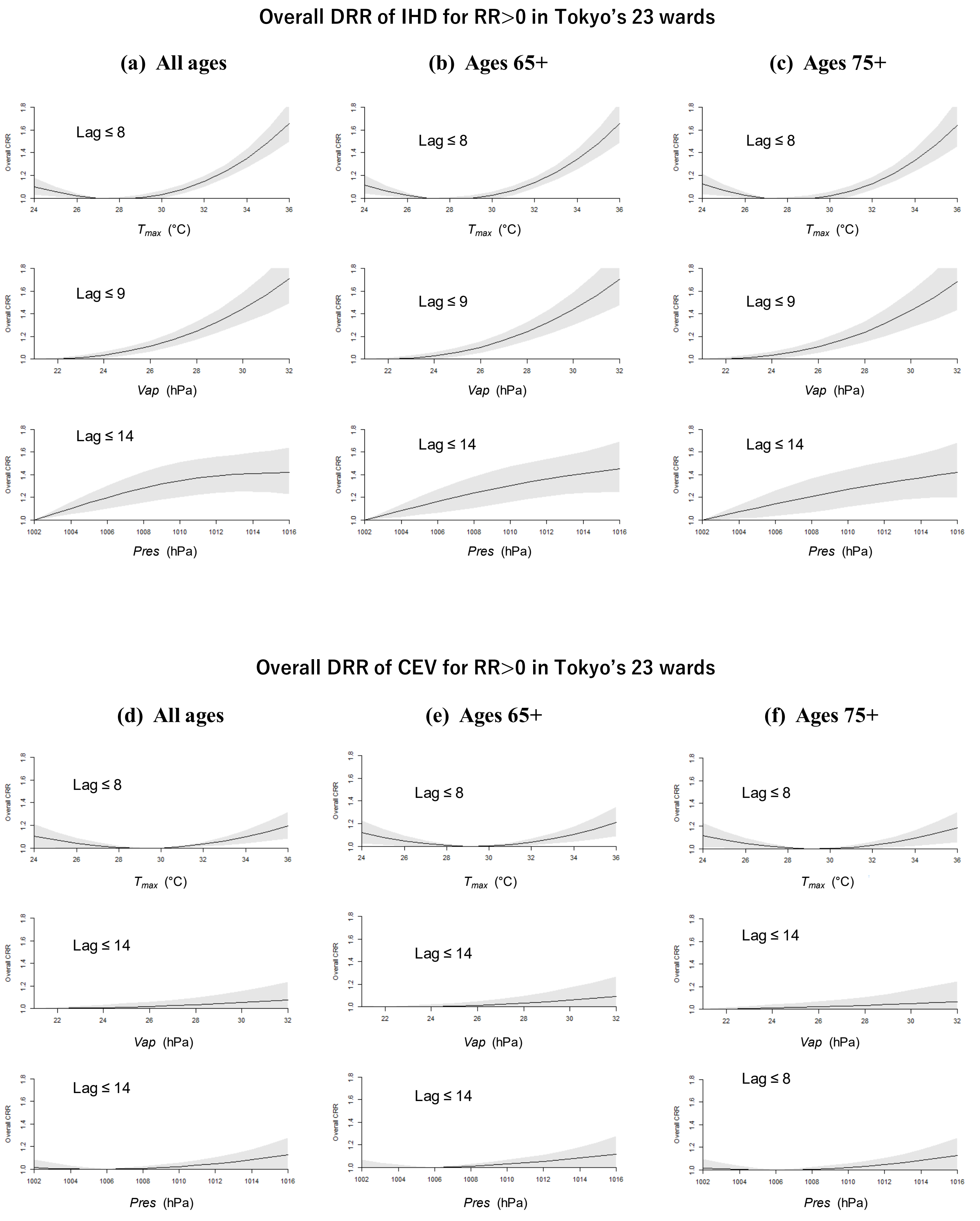

Supplementary Figure S3. Results of overall cumulative DRR using the DLNM in Tokyo’s 23 wards. (a)–(c) overall DRR of IHD and (d)–(f) overall DRR of CEV for (a)(d) all ages, (b)(e) ages 65+, and (c)(f) ages 75+ years. Each panel indicates weather variables (T_{max}, *Vap*, and *Pres*) versus DRR.

DRR of IHD to T_{max} in Tokyo's 23 wards

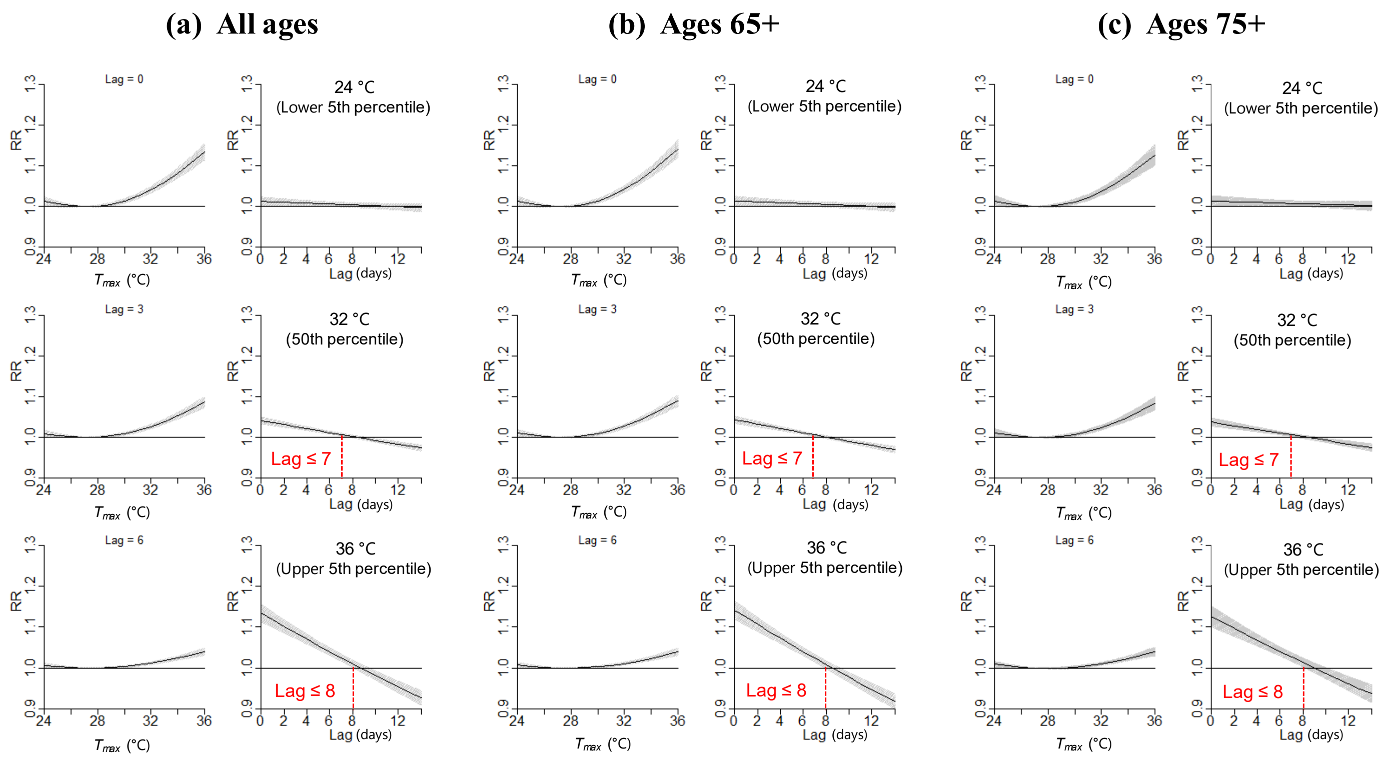

DRR of IHD to *Vap* in Tokyo's 23 wards

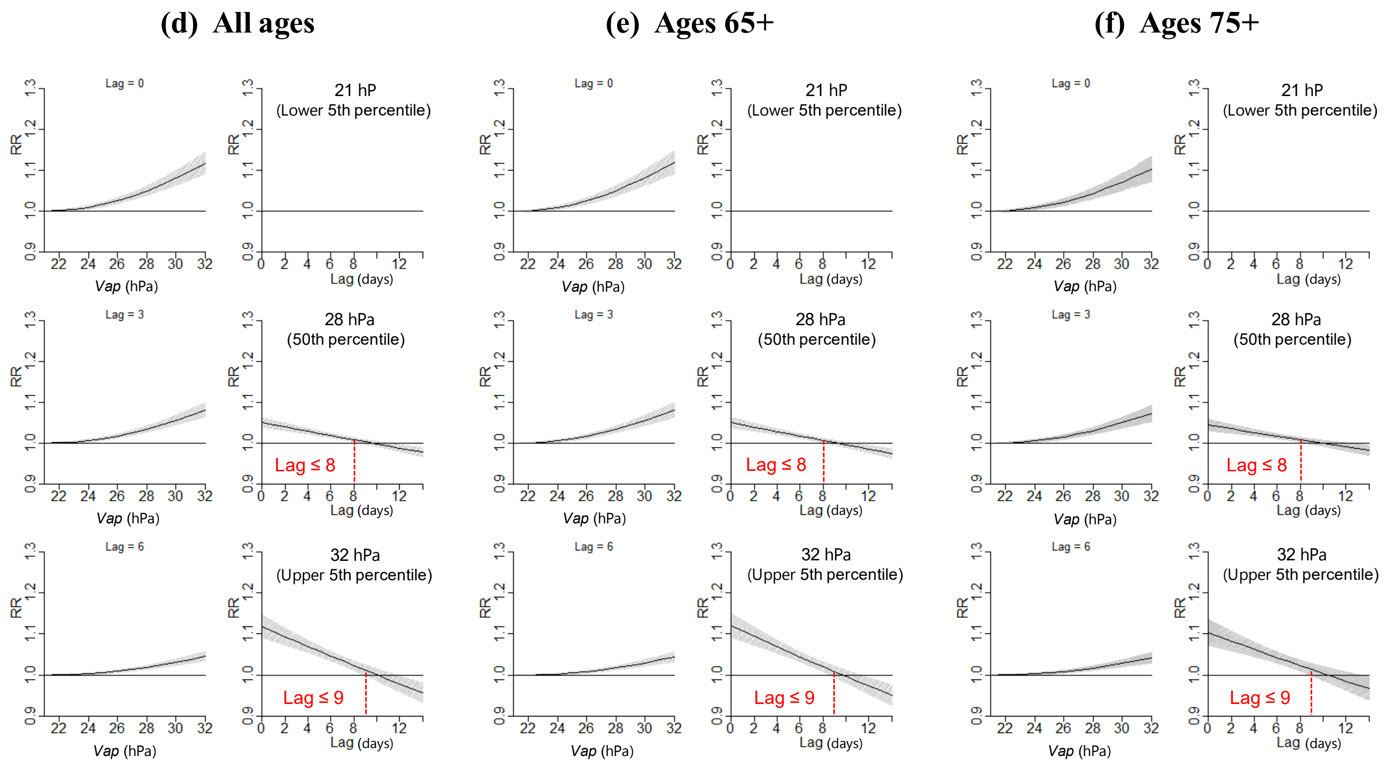

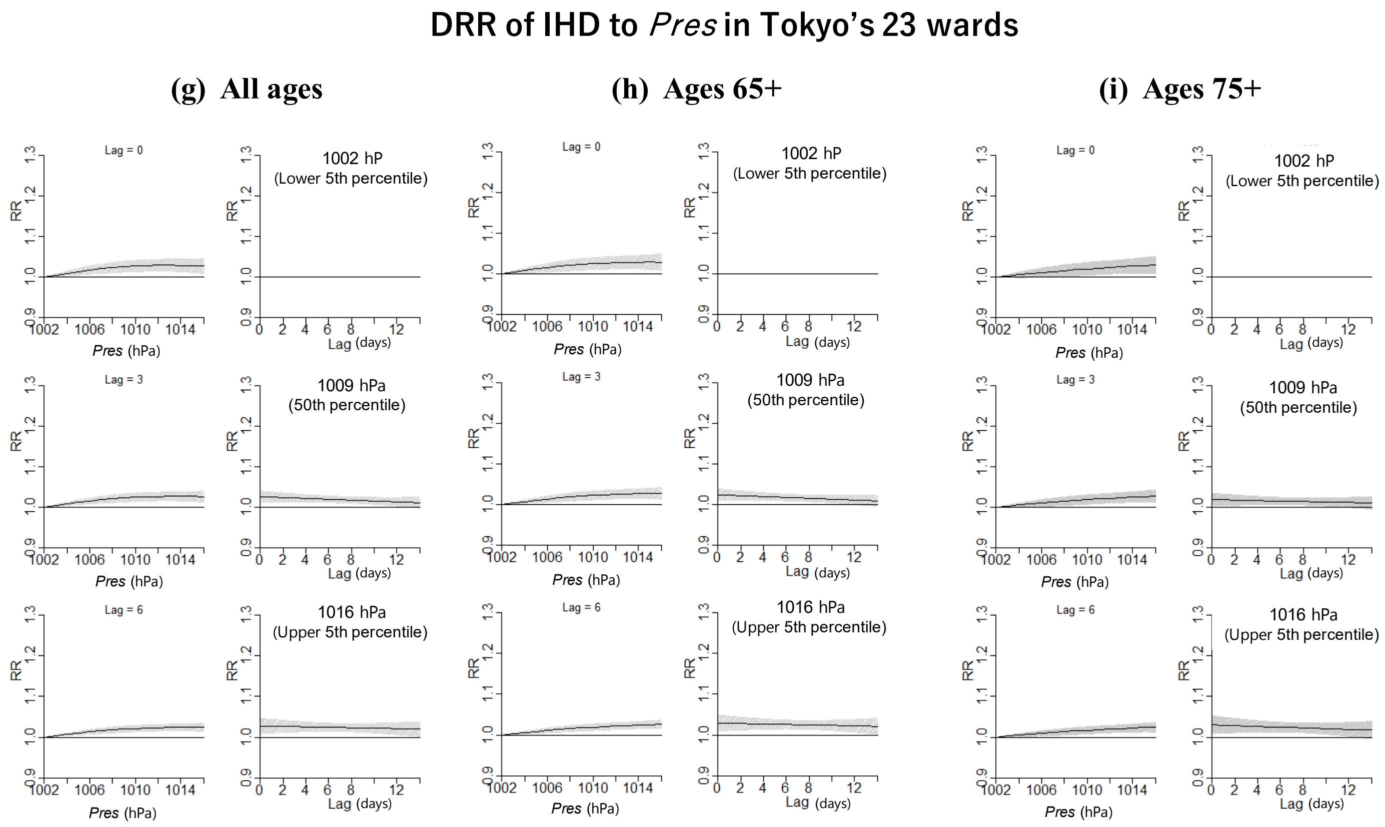

Supplementary Figure S4. Results of lag analysis using the DLNM in Tokyo's 23 wards. The DRR of IHD to (a)–(c) T_{max}, (d)–(f) *Vap*, and (d)–(f) *Pres* were divided into (a)(d)(g) all ages, and (b)(e)(h) ages 65+, and (c)(f)(i) ages 75+. Each panel indicates (left side in the panel) weather variables versus DRR at lags of 0, 3, 6 days and (right side in the panel) lag days versus DRR at the lower 5th, 50th, and upper 5th (95th) percentiles of weather variables.

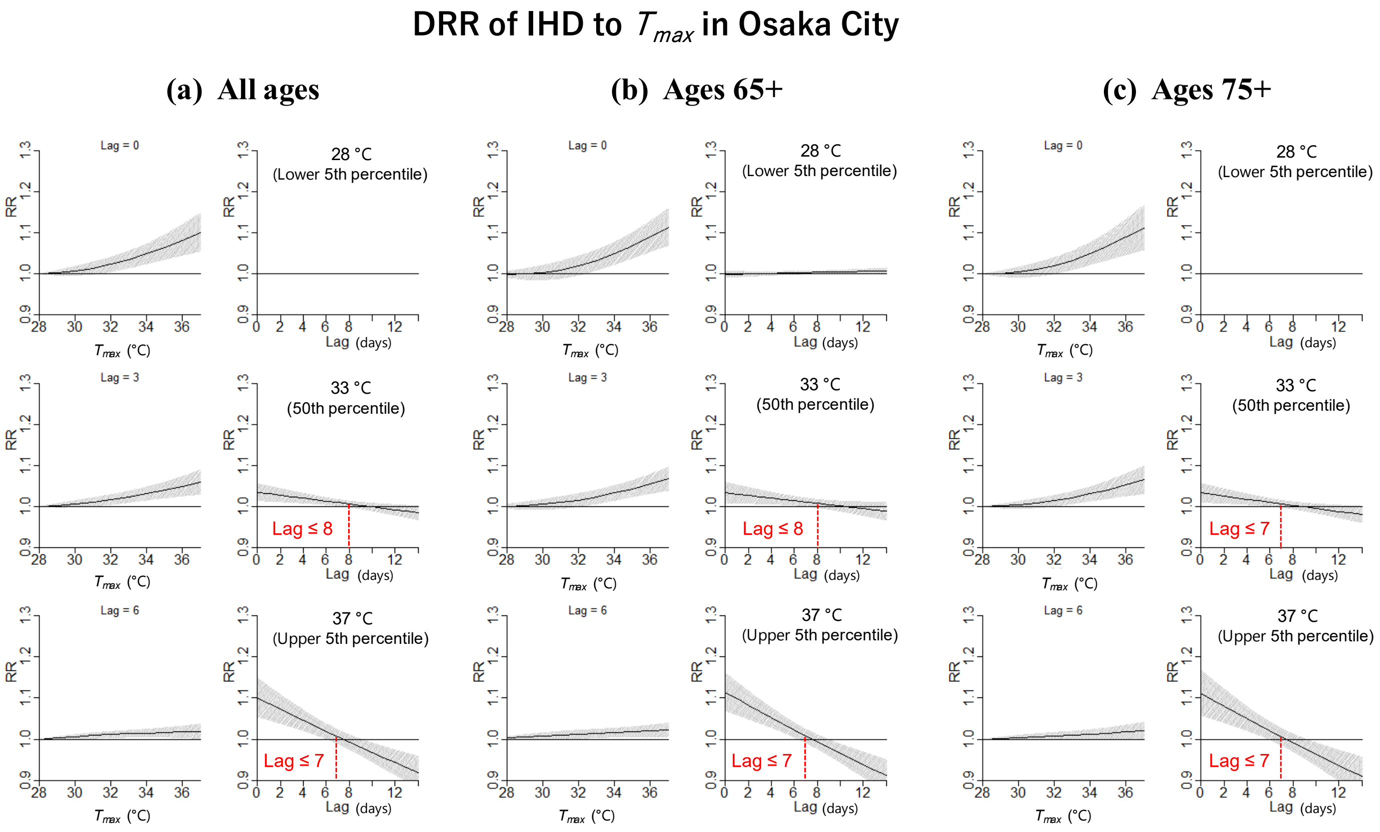
DRR of IHD to Tmax in Osaka City
(a) All ages
(b) Ages 65+
(c) Ages 75+
Lag = 0
Lag = 3
Lag = 6
28 °C
(Lower 5th percentile)
33 °C
(50th percentile)
37 °C
(Upper 5th percentile)
Lag ≤ 8
Lag ≤ 7
RR
Tmax (°C)
Lag (days)

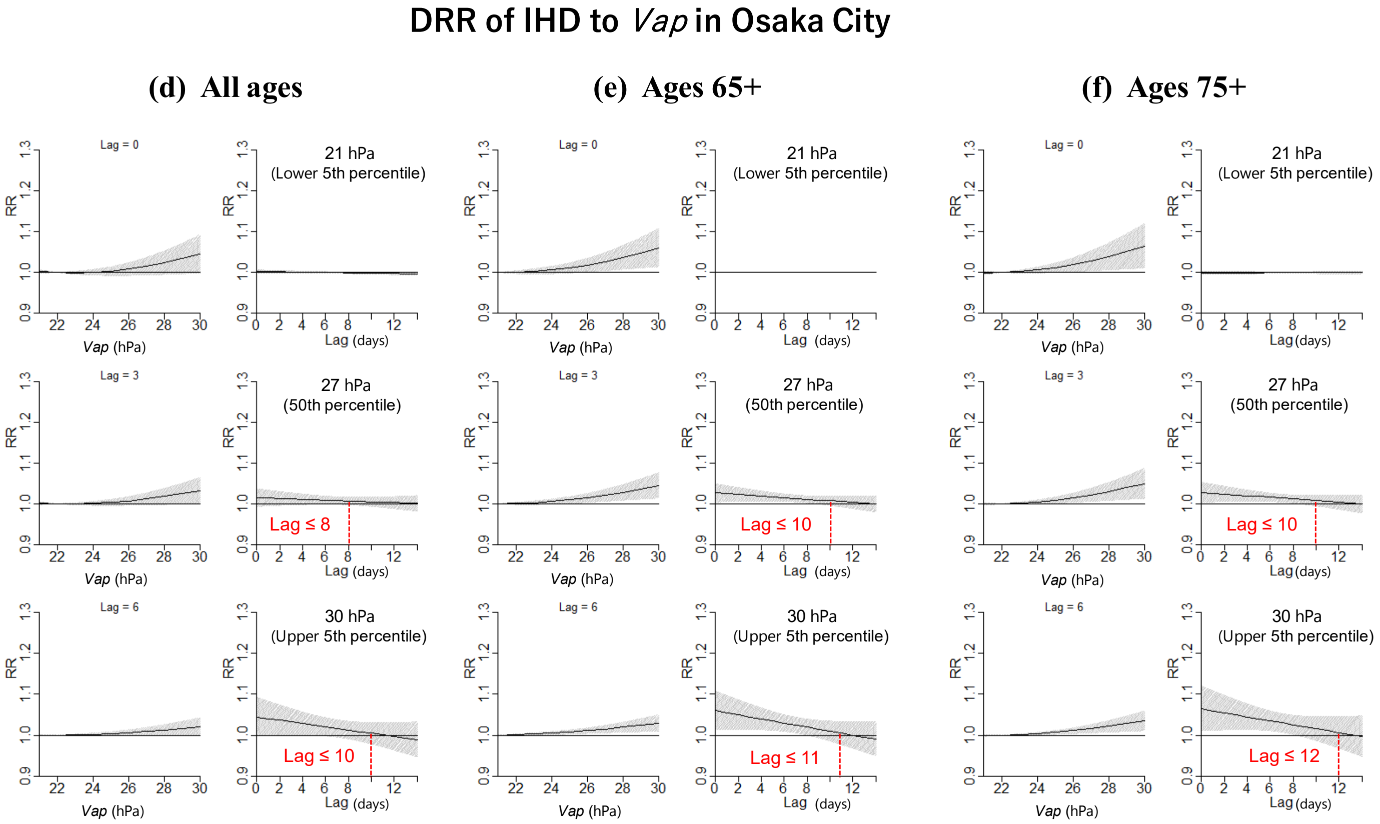
DRR of IHD to Vap in Osaka City
(d) All ages
(e) Ages 65+
(f) Ages 75+
Lag = 0
Lag = 3
Lag = 6
21 hPa
(Lower 5th percentile)
27 hPa
(50th percentile)
30 hPa
(Upper 5th percentile)
Lag ≤ 8
Lag ≤ 10
Lag ≤ 11
Lag ≤ 12
RR
Vap (hPa)
Lag (days)

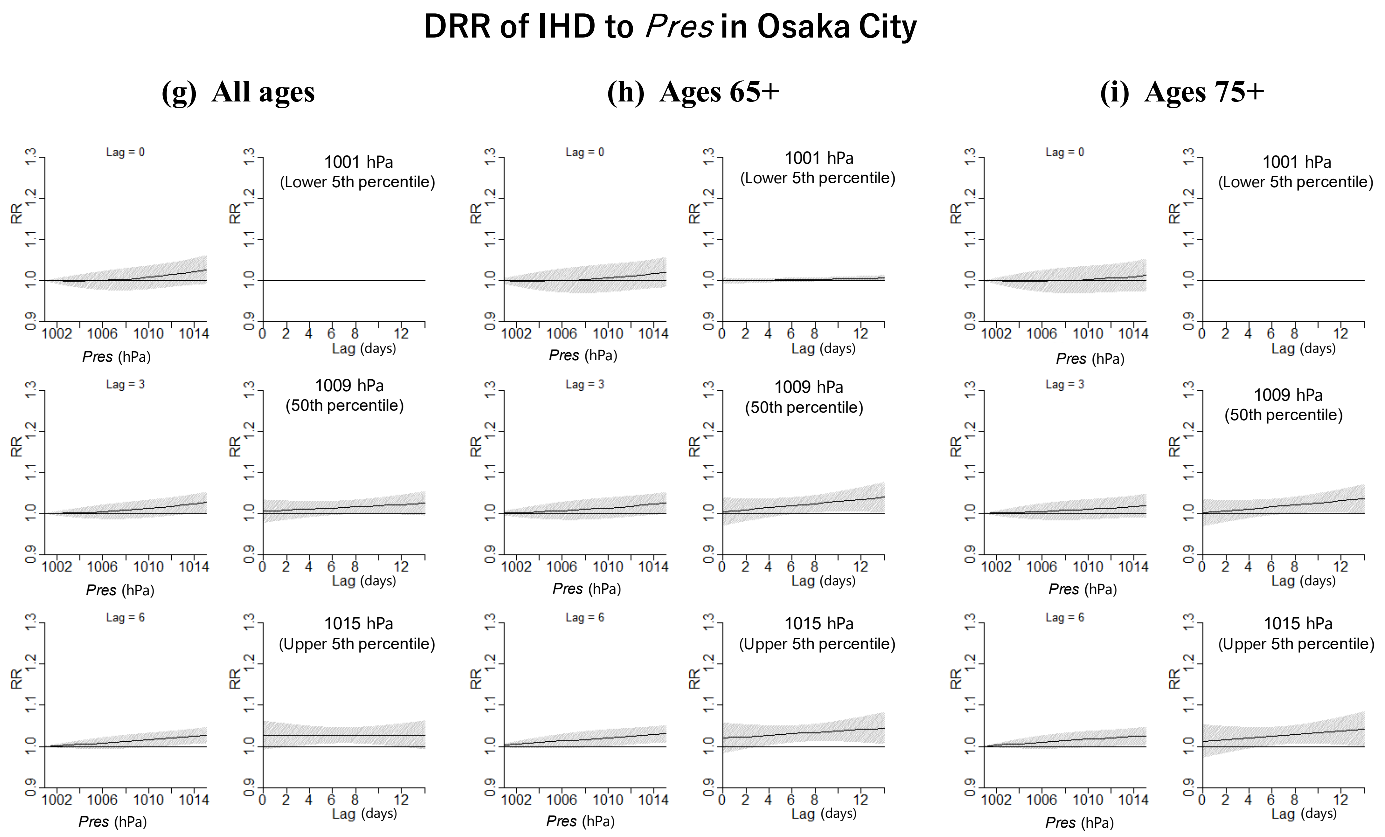

Supplementary Figure S5. Results of lag analysis using the DLNM in Osaka City. The DRR of IHD to (a)–(c) T_{max}, (d)–(f) *Vap*, and (d)–(f) *Pres* were divided into (a)(d)(g) all ages, and (b)(e)(h) ages 65+, and (c)(f)(i) ages 75+. Each panel indicates (left side in the panel) weather variables versus DRR at lags of 0, 3, 6 days and (right side in the panel) lag days versus DRR at the lower 5th, 50th, and upper 5th (95th) percentiles of weather variables.

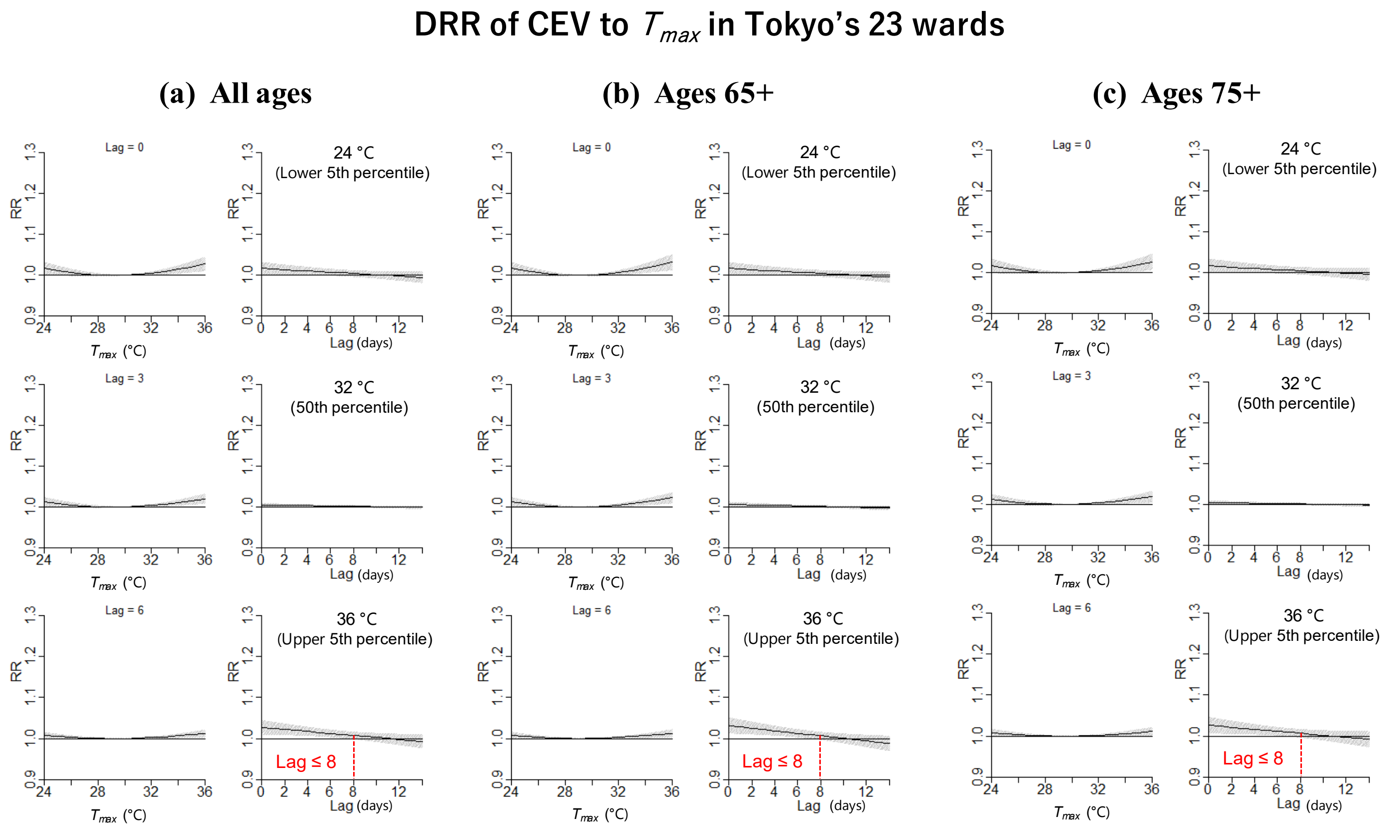
DRR of CEV to T_{max} in Tokyo's 23 wards
(a) All ages
(b) Ages 65+
(c) Ages 75+
Lag = 0
Lag = 3
Lag = 6
24 °C
(Lower 5th percentile)
32 °C
(50th percentile)
36 °C
(Upper 5th percentile)
Lag ≤ 8
RR
T_{max} (°C)
Lag (days)

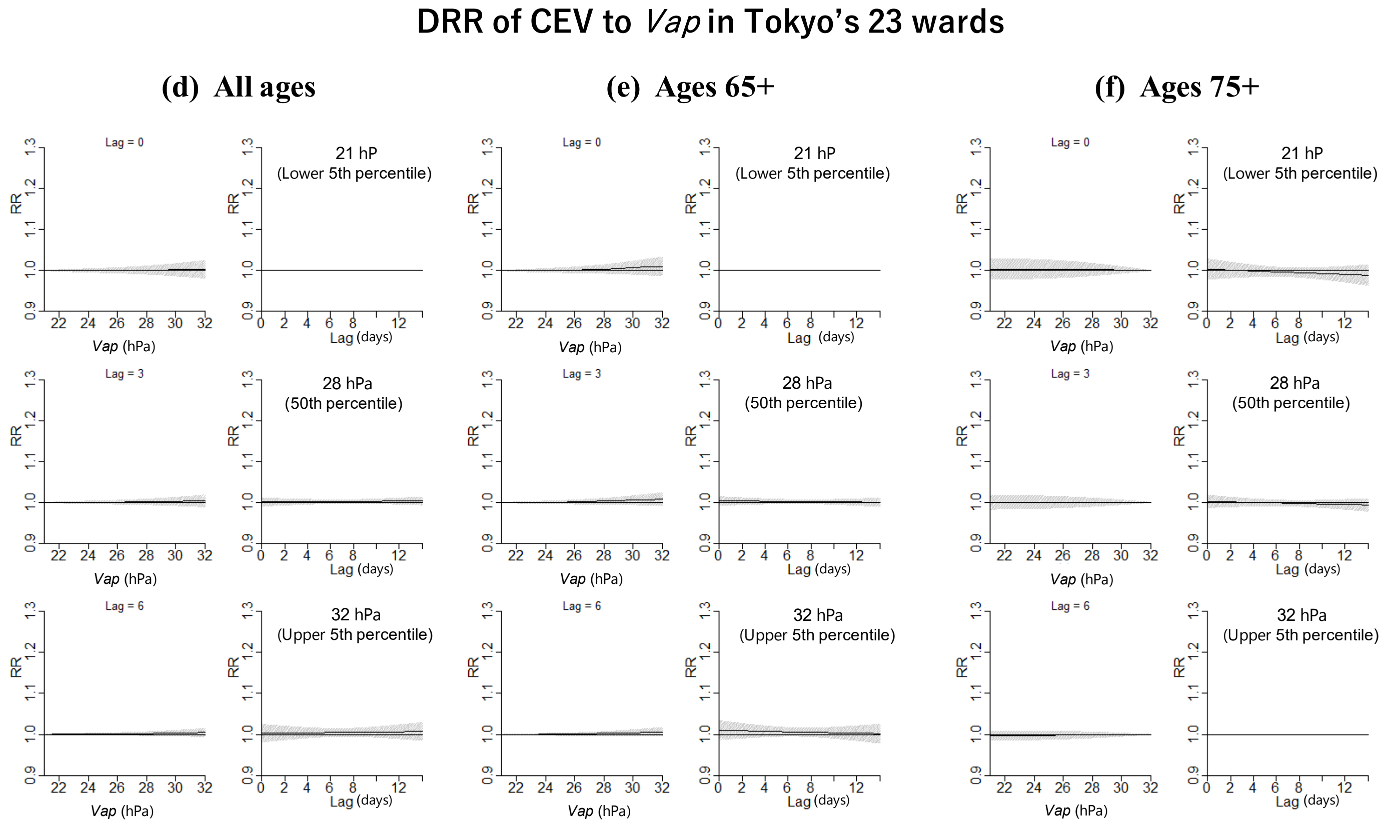
DRR of CEV to *Vap* in Tokyo's 23 wards
(d) All ages
(e) Ages 65+
(f) Ages 75+
Lag = 0
Lag = 3
Lag = 6
21 hP
(Lower 5th percentile)
28 hPa
(50th percentile)
32 hPa
(Upper 5th percentile)
RR
Vap (hPa)
Lag (days)

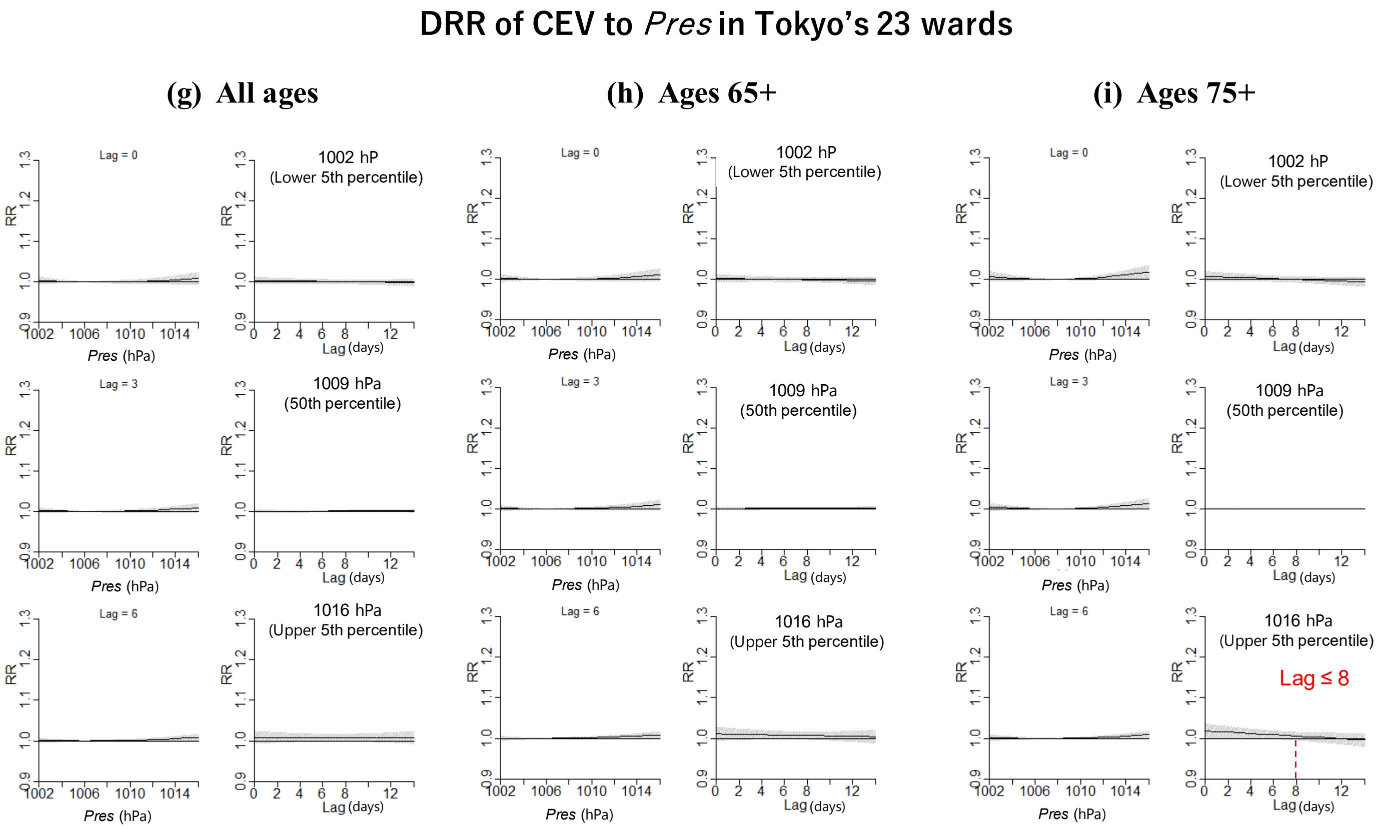

Supplementary Figure S6. Results of lag analysis using the DLNM in Tokyo's 23 wards. The DRR of CEV to (a)–(c) T_{max}, (d)–(f) *Vap*, and (d)–(f) *Pres* were divided into (a)(d)(g) all ages, and (b)(e)(h) ages 65+, and (c)(f)(i) ages 75+. Each panel indicates (left side in the panel) weather variables versus DRR at lags of 0, 3, 6 days and (right side in the panel) lag days versus DRR at the lower 5th, 50th, and upper 5th (95th) percentiles of weather variables.

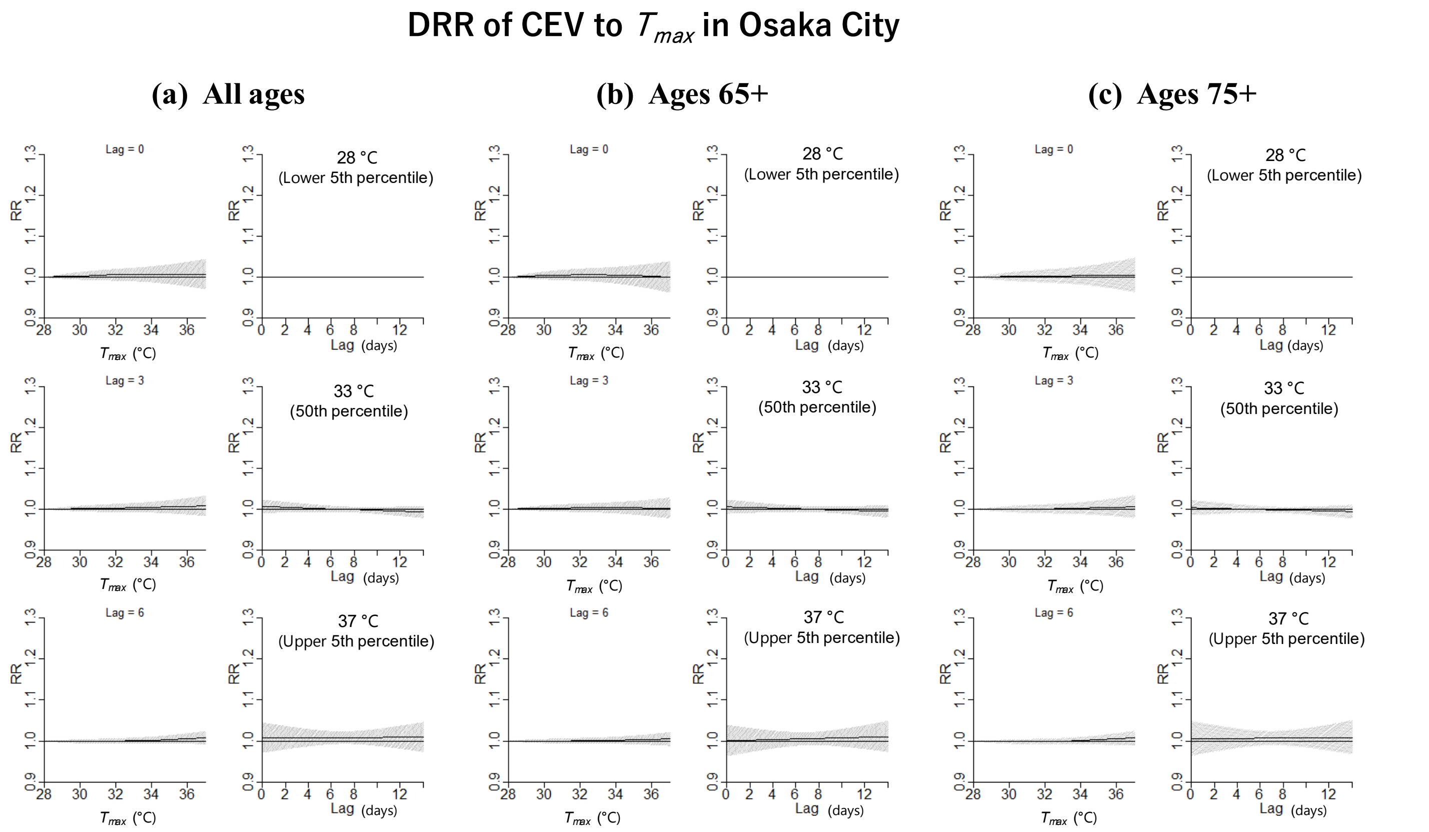
DRR of CEV to T_{max} in Osaka City
(a) All ages
(b) Ages 65+
(c) Ages 75+
Lag = 0
Lag = 3
Lag = 6
28 °C
(Lower 5th percentile)
33 °C
(50th percentile)
37 °C
(Upper 5th percentile)
RR
T_{max} (°C)
Lag (days)

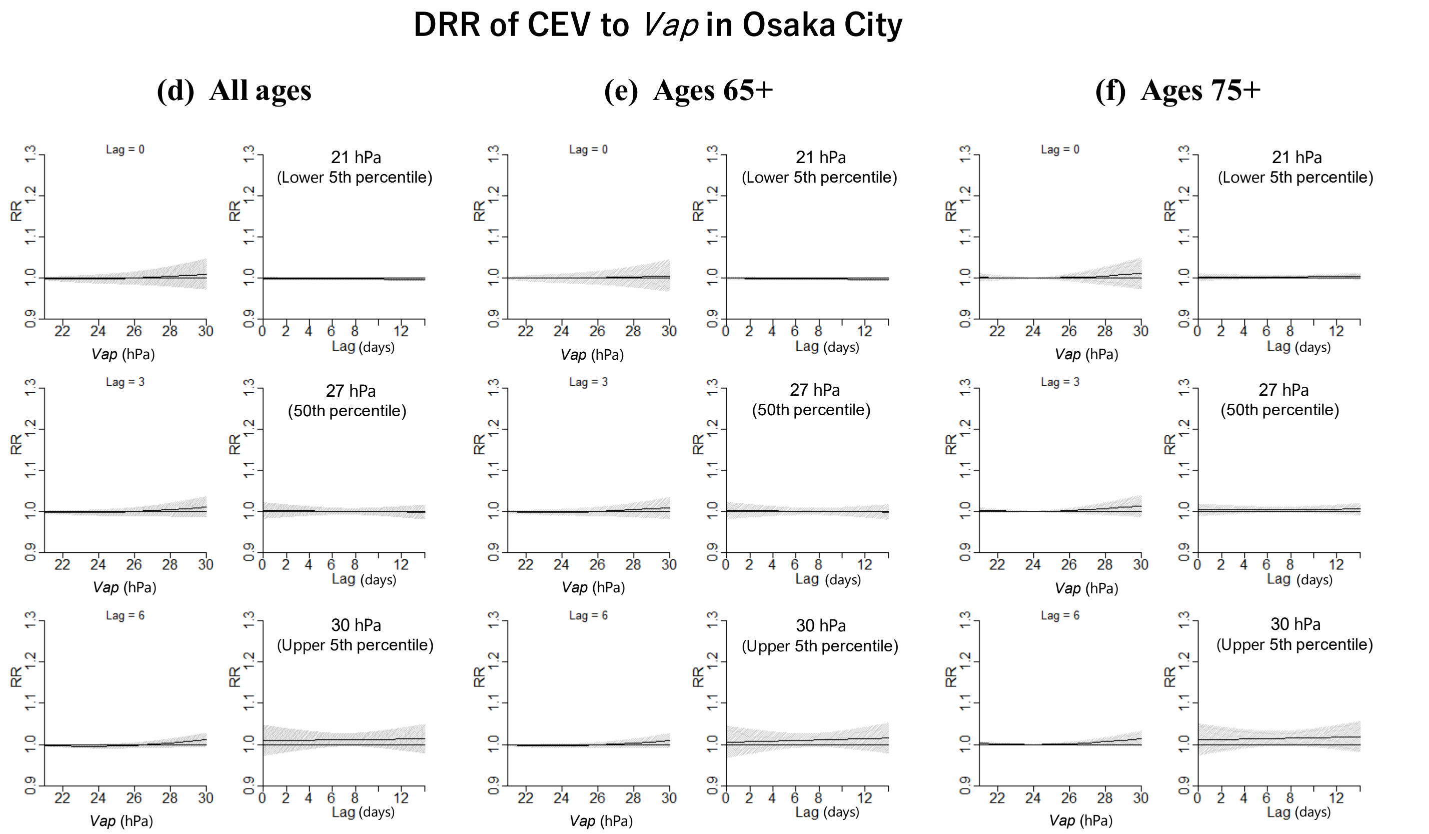
DRR of CEV to Vap in Osaka City
(d) All ages
(e) Ages 65+
(f) Ages 75+
Lag = 0
Lag = 3
Lag = 6
21 hPa
(Lower 5th percentile)
27 hPa
(50th percentile)
30 hPa
(Upper 5th percentile)
RR
Vap (hPa)
Lag (days)

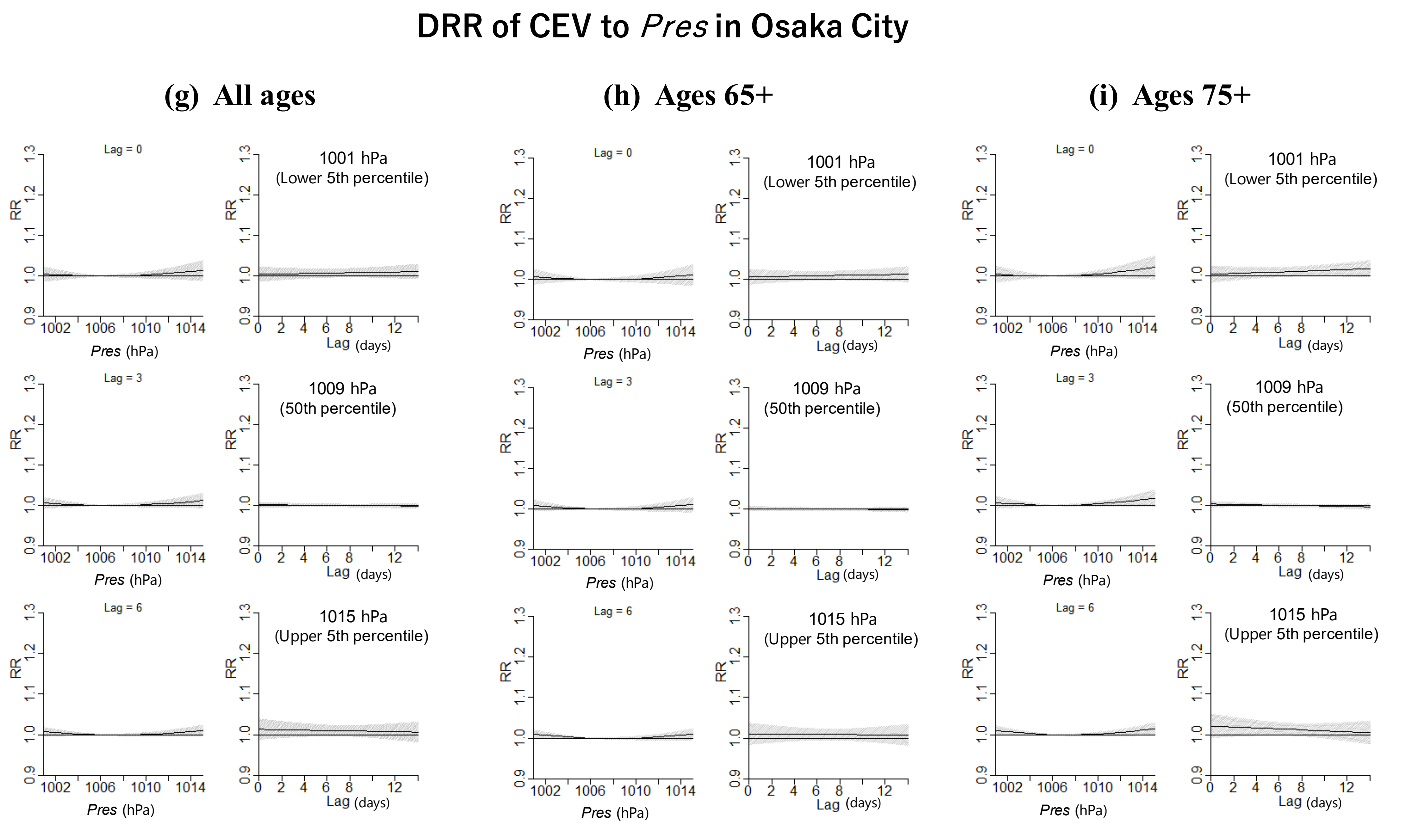

Supplementary Figure S7. Results of lag analysis using the DLNM in Osaka City. The DRR of CEV to (a)–(c) T_{max}, (d)–(f) *Vap*, and (d)–(f) *Pres* were divided into (a)(d)(g) all ages, and (b)(e)(h) ages 65+, and (c)(f)(i) ages 75+. Each panel indicates (left side in the panel) weather variables versus DRR at lags of 0, 3, 6 days and (right side in the panel) lag days versus DRR at the lower 5th, 50th, and upper 5th (95th) percentiles of weather variables.

(a) ML results of IHD mortality at ages 65+ in Tokyo's 23 wards

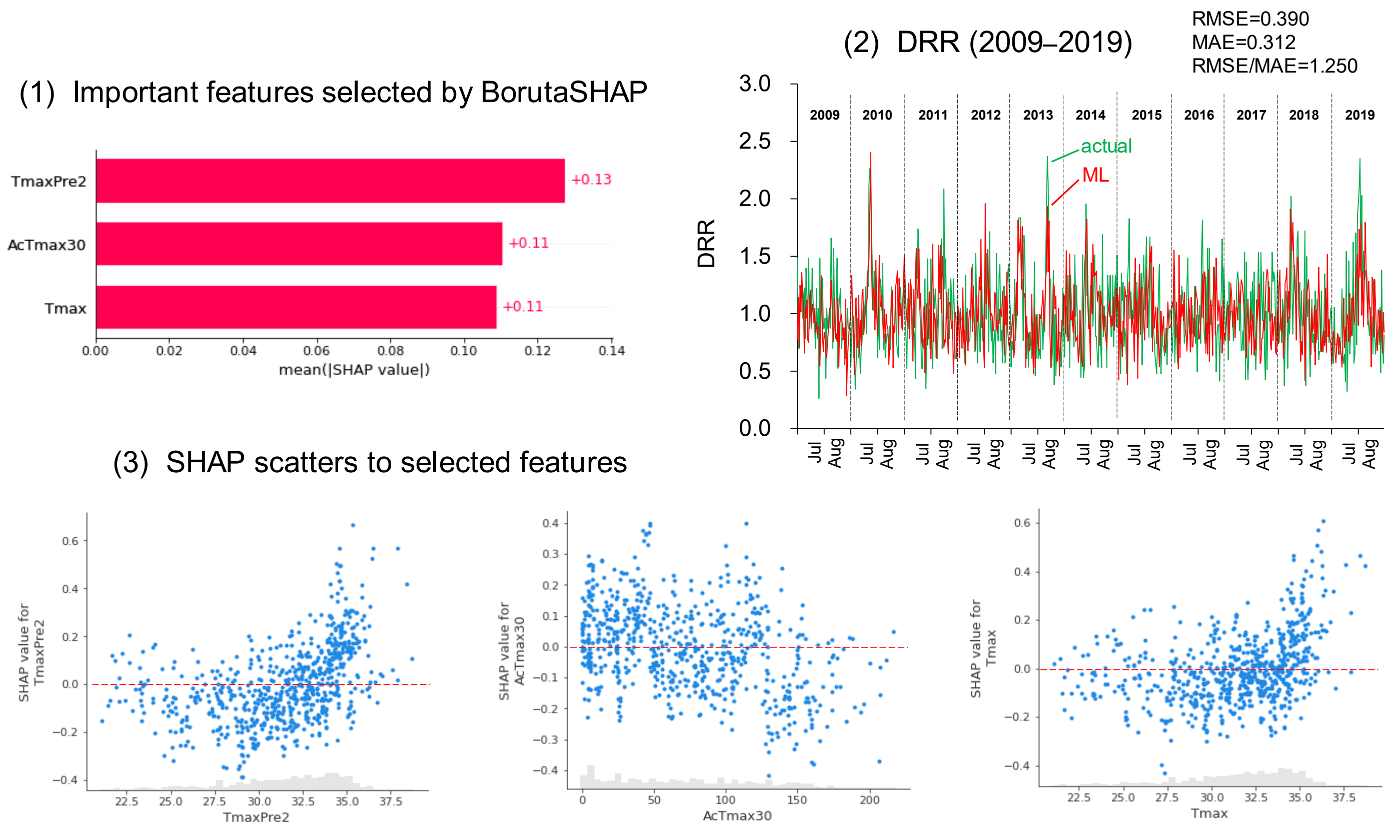

(b) ML results of IHD mortality at ages 75+ in Tokyo's 23 wards

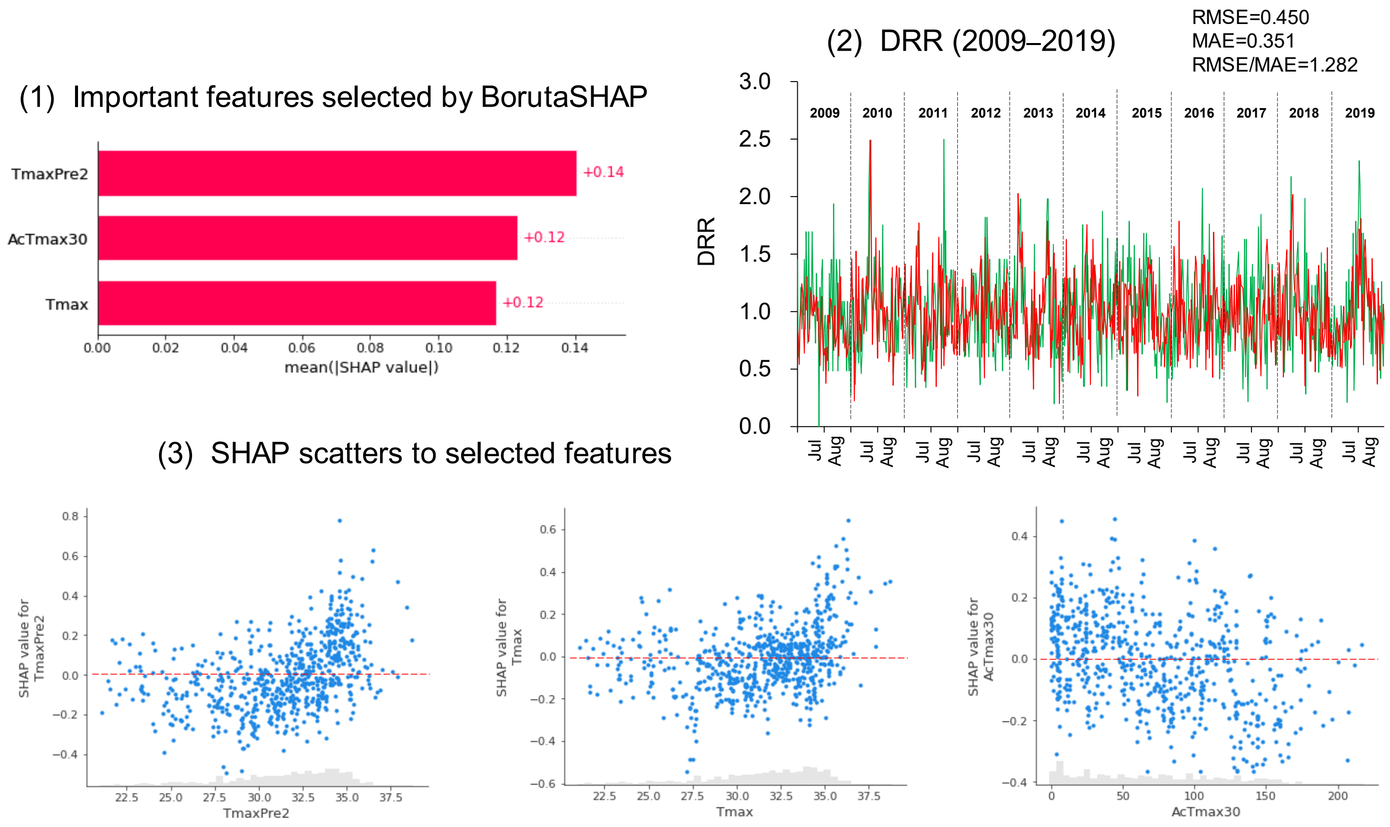

(c) ML results of IHD mortality at all ages in Osaka City

(1) Important feature selected by BorutaSHAP

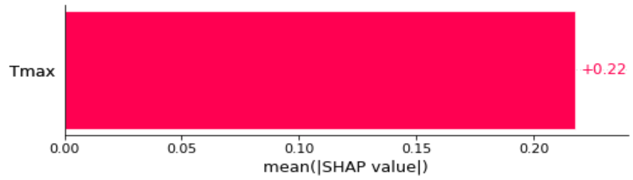

(2) DRR (2009–2019)

RMSE=0.595
MAE=0.462
RMSE/MAE=1.288

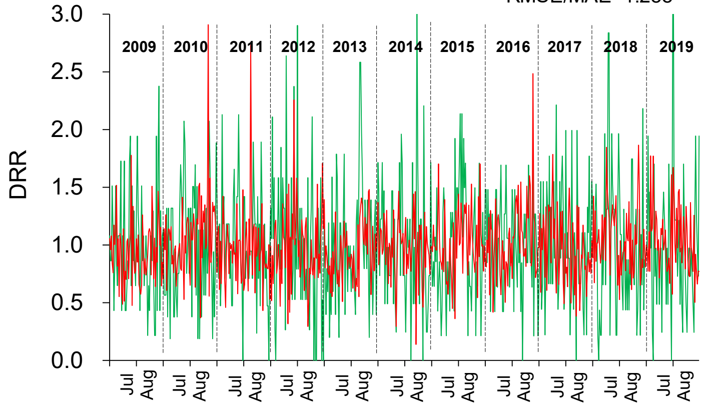

(3) SHAP scatters to selected feature

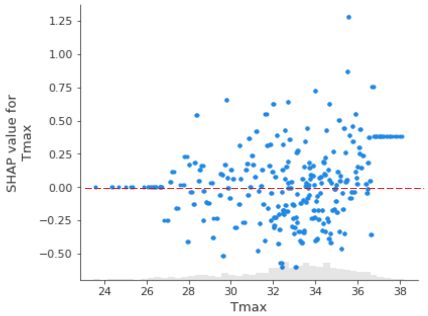

(d) ML results of IHD mortality at ages 65+ in Osaka City

(1) Selected features by BorutaShap

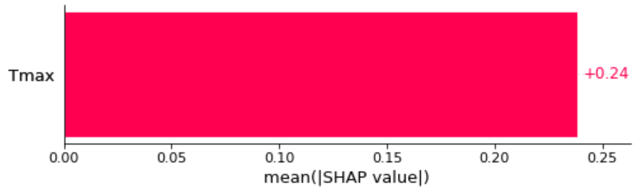

(2) DRR (2009–2019)

RMSE=0.631
MAE=0.498
RMSE/MAE=1.267

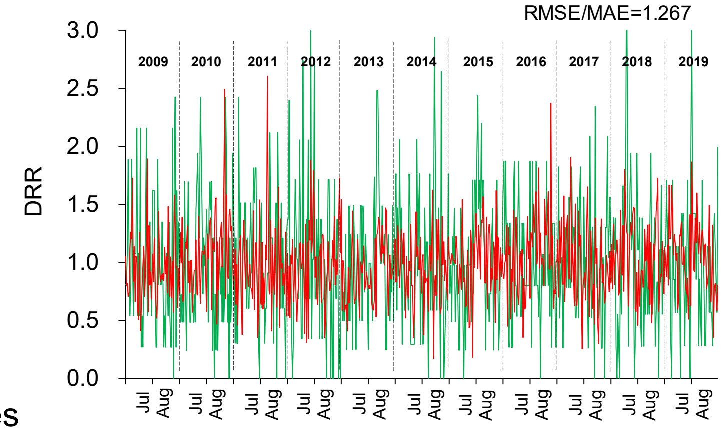

(3) SHAP scatters to selected features

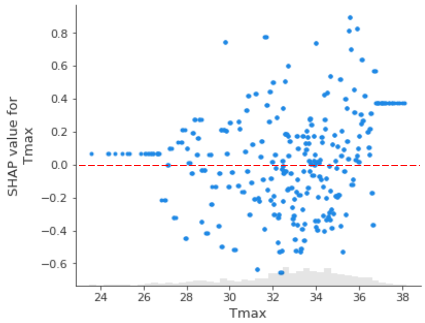

(e) ML results of IHD mortality at ages 75+ in Osaka City

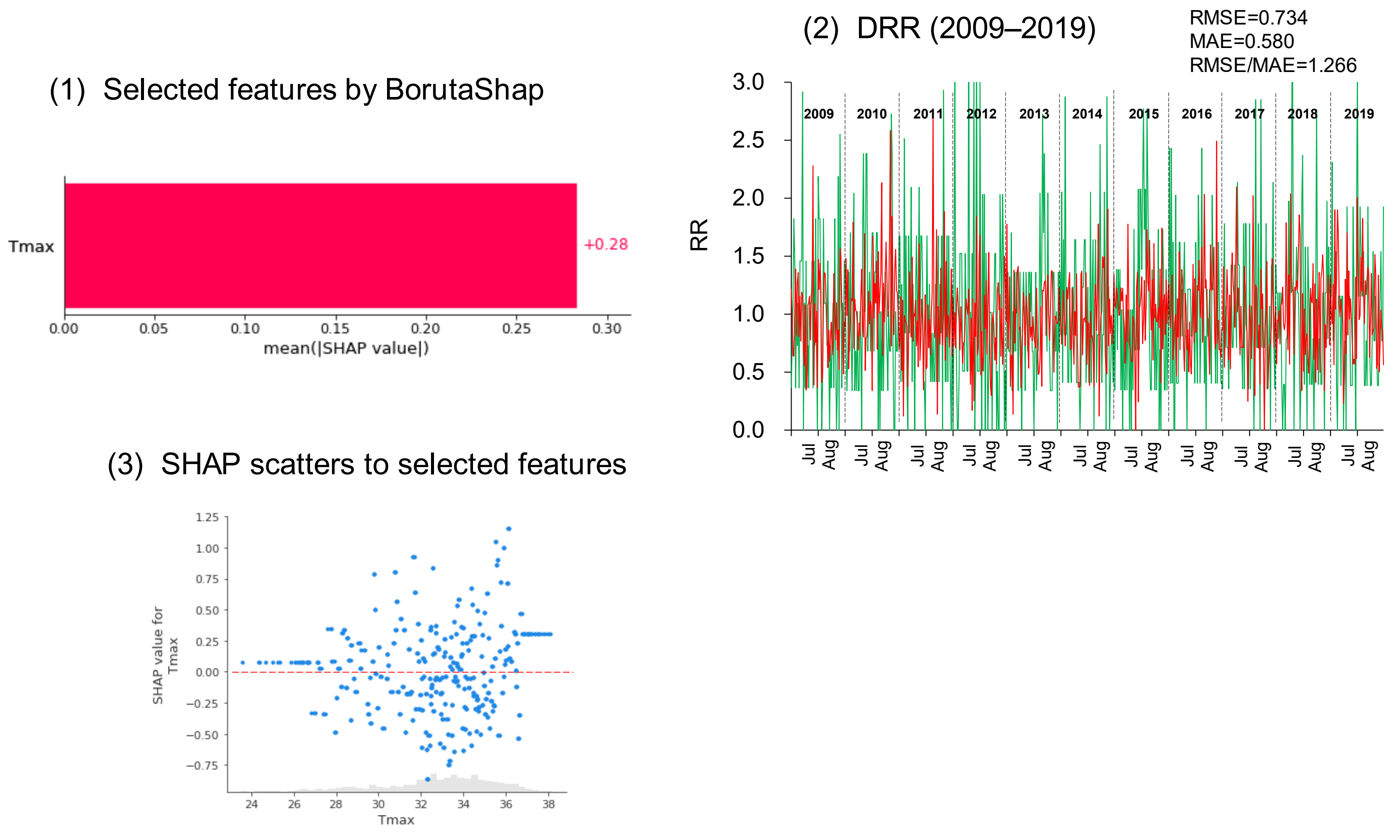

Supplementary Figure S8. ML hindcast of IHD mortality during summer at (a) ages 65+ and (b) ages 75+ years in Tokyo's 23 wards, and (c) all ages, (d) ages 65+, and (e) ages 75+ years in Osaka City (2009–2019). (1) Important features selected using BorutaSHAP, (2) comparison between simulated and actual DRR, and (3) relationships between SHAP values and the selected important features. ML, machine learning; BorutaSHAP, Boruta SHapley Additive exPlanations; DRR, daily relative risk; IHD, ischaemic heart disease; CEV, cerebrovascular disease; RMSE, root mean square error; MAE, mean absolute error.

(a) ML results of CEV mortality at ages 65+ in Tokyo's 23 wards

(1) Selected features by BorutaShap

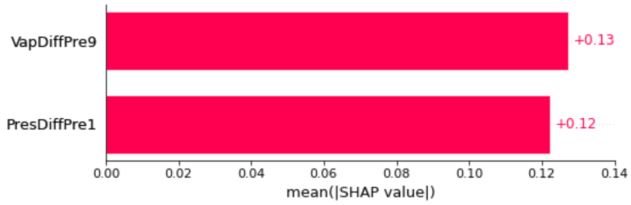

(2) DRR (2009–2019)

RMSE=0.351
MAE=0.281
RMSE/MAE=1.248

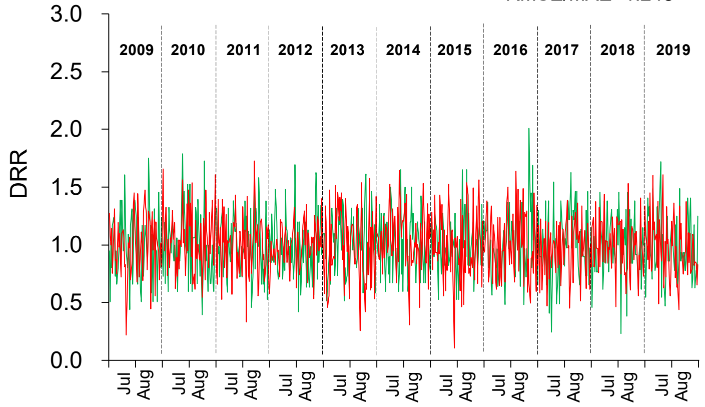

(3) SHAP scatters to selected features

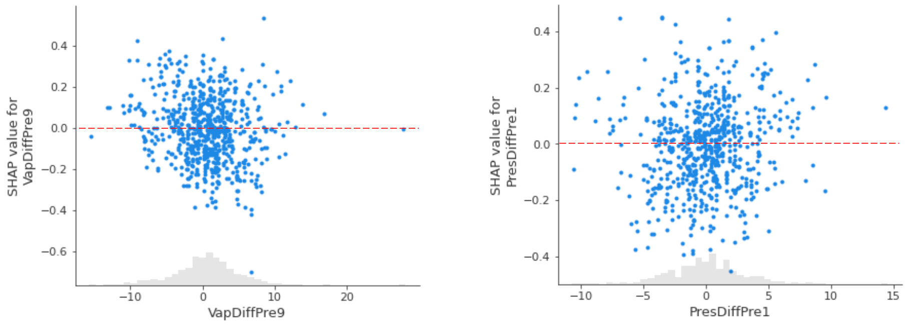

(b) ML results of CEV mortality at ages 75+ in Tokyo's 23 wards

(1) Selected features by BorutaShap

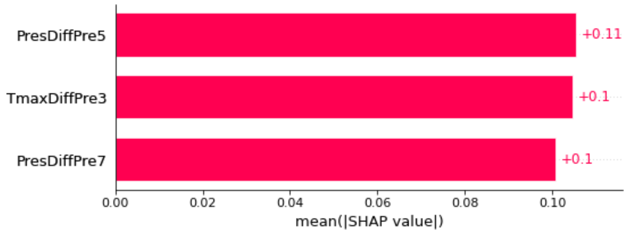

(2) DRR (2009–2019)

RMSE=0.374
MAE=0.296
RMSE/MAE=1.263

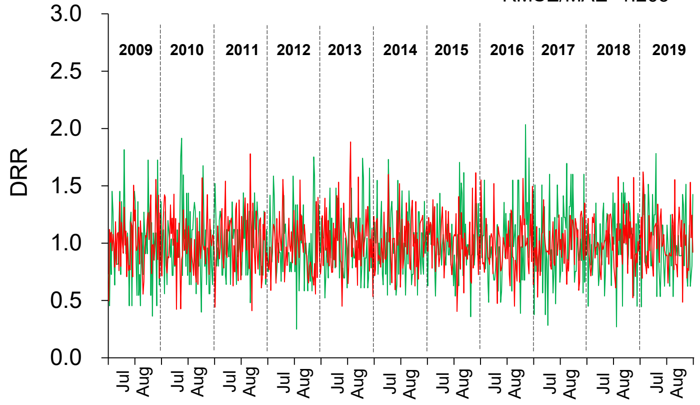

(3) SHAP scatters to selected features

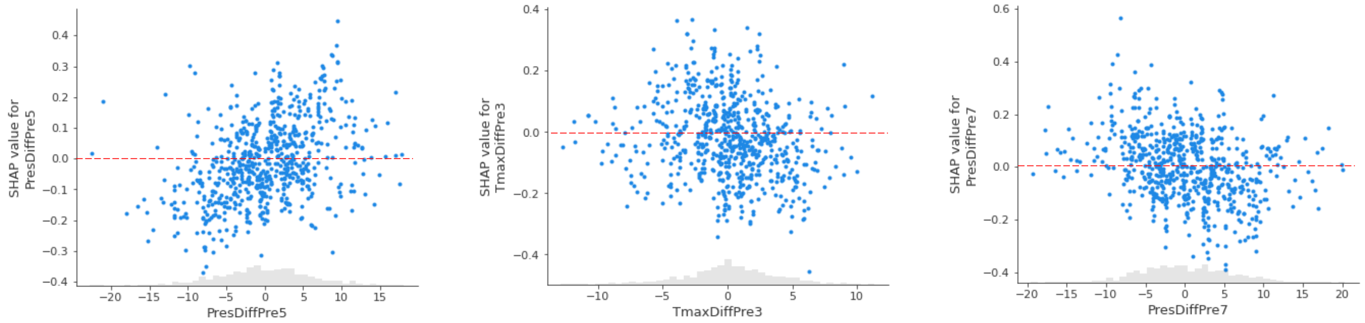

(c) ML results of CEV mortality at all ages in Osaka City

(1) Selected features by BorutaShap

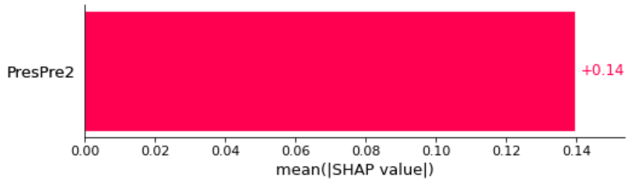

(2) DRR (2009–2019)

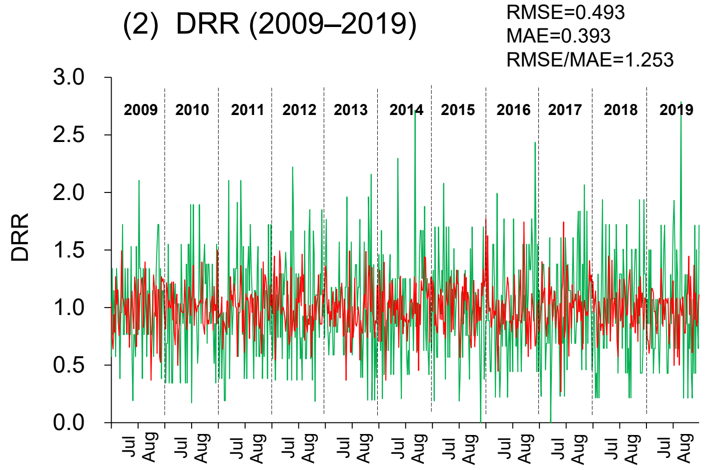

(3) SHAP scatters to selected features

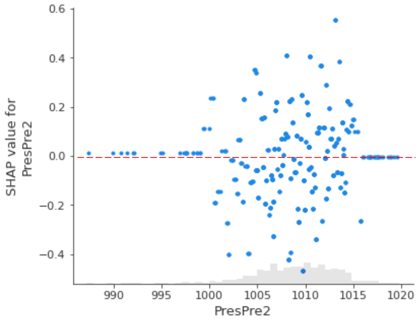

(d) ML results of CEV mortality at ages 65+ in Osaka City

(2) DRR (2009–2019)

(1) Selected features by BorutaShap

No selected feature importance

(3) SHAP scatters to selected features

(e) ML results of CEV mortality at ages 75+ in Osaka City

(1) Selected features by BorutaShap

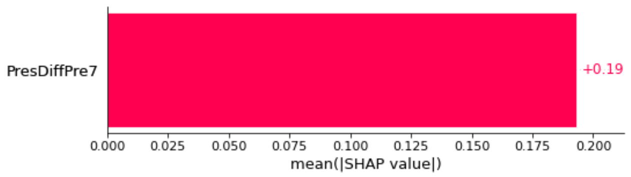

(2) DRR (2009–2019)

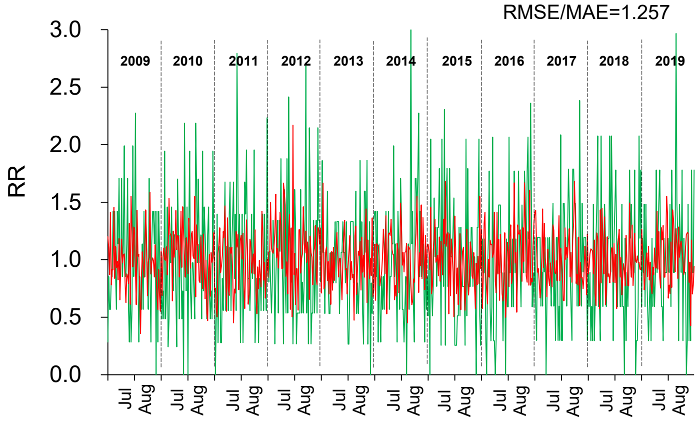

(3) SHAP scatters to selected features

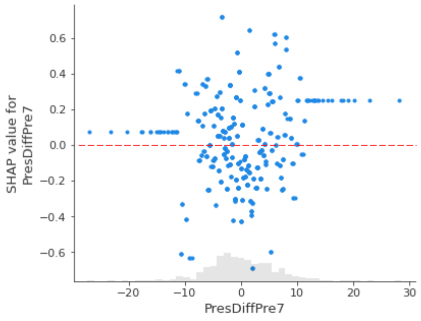

Supplementary Figure S9. ML hindcast of CEV mortality during summer at (a) ages 65+ and (b) ages 75+ years in Tokyo's 23 wards, and (c) all ages, (d) ages 65+, and (e) ages 75+ years in Osaka City (2009–2019). (1) Important features selected using BorutaSHAP, (2) comparison between simulated and actual DRR, and (3) relationships between SHAP values and the selected important features. The result of (d) indicates no selected feature importance from BorutaSHAP.

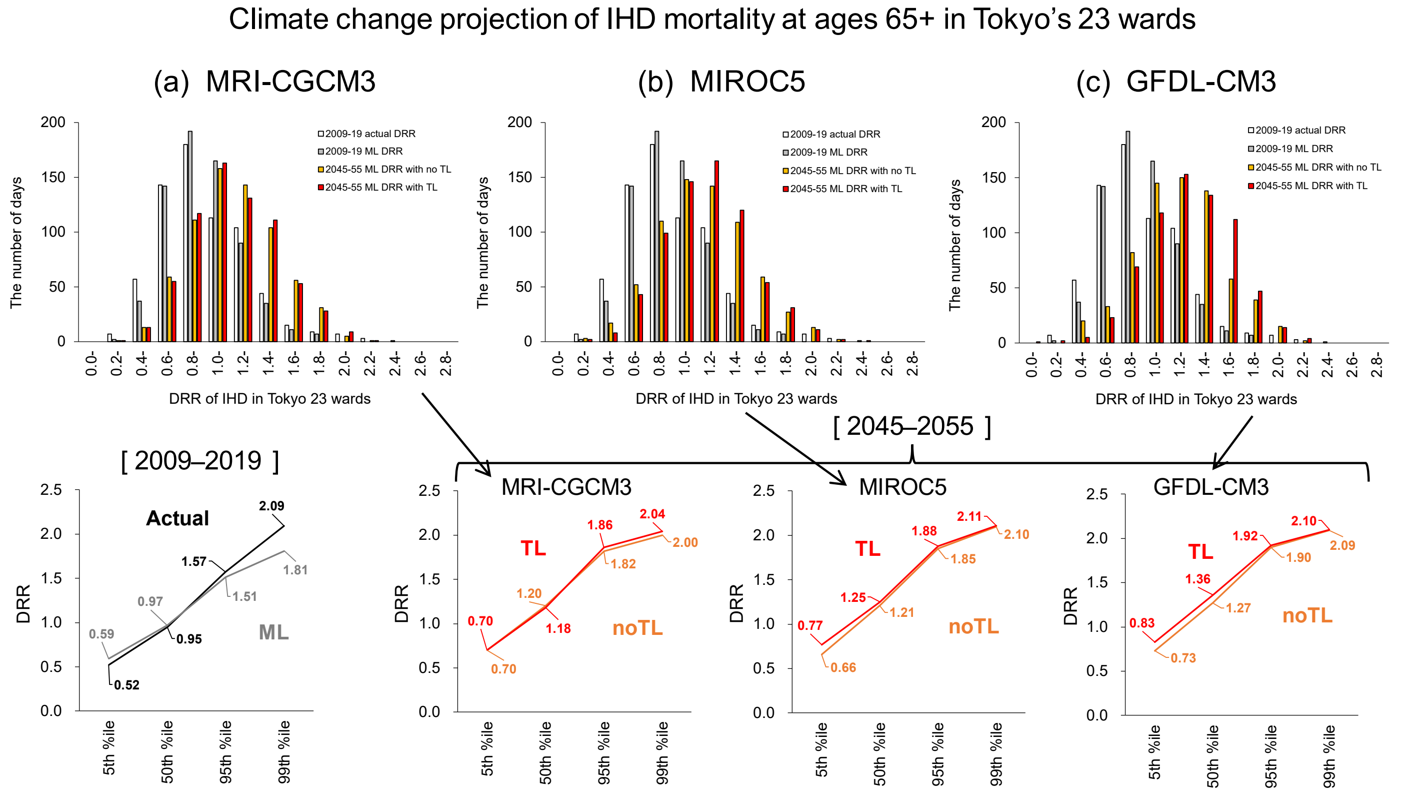

Supplementary Figure S10. Frequency distributions (upper panels) and percentiles (lower panels) of the DRR of IHD at ages 65+ years in Tokyo's 23 wards. Against actual and ML results in 2009–2019, the DRR change was estimated using climate projections of (a) MRI-CGCM3, (b) MIROC5, and (c) GFDL-CM3 under the condition RCP8.5. "no TL" and "TL" indicate ML cases incorporating no TL and incorporating TL, respectively. ML, machine learning; DRR, daily relative risk; IHD, ischaemic heart disease; TL, transfer learning.

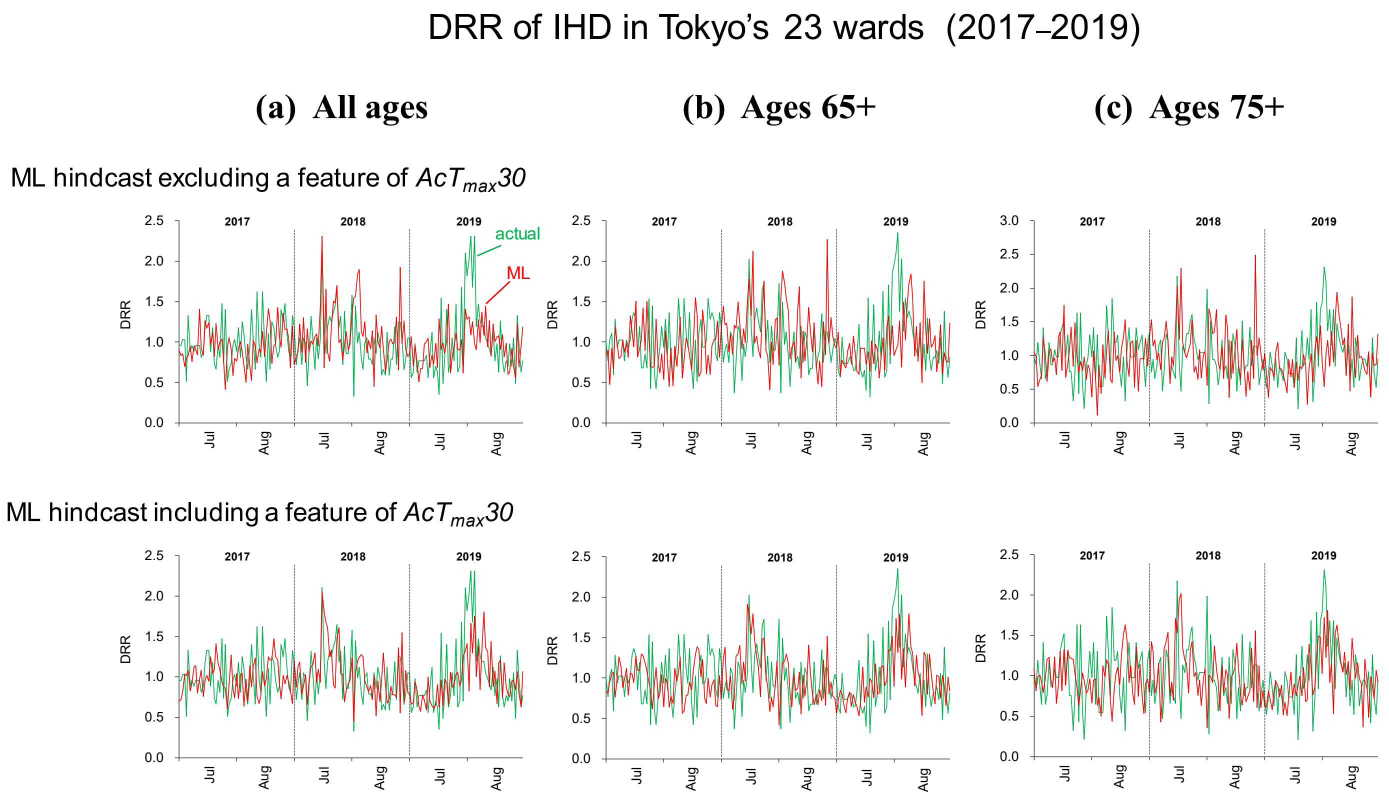

Supplementary Figure S11. ML hindcast of IHD mortality during summer at (a) all ages, (b) ages 65+, and (c) ages 75+ years in Tokyo's 23 wards (2017–2019). Comparisons between simulated and actual DRR are indicated as the results (upper panels) excluding and (lower panels) including a feature of $AcT_{max}30$.

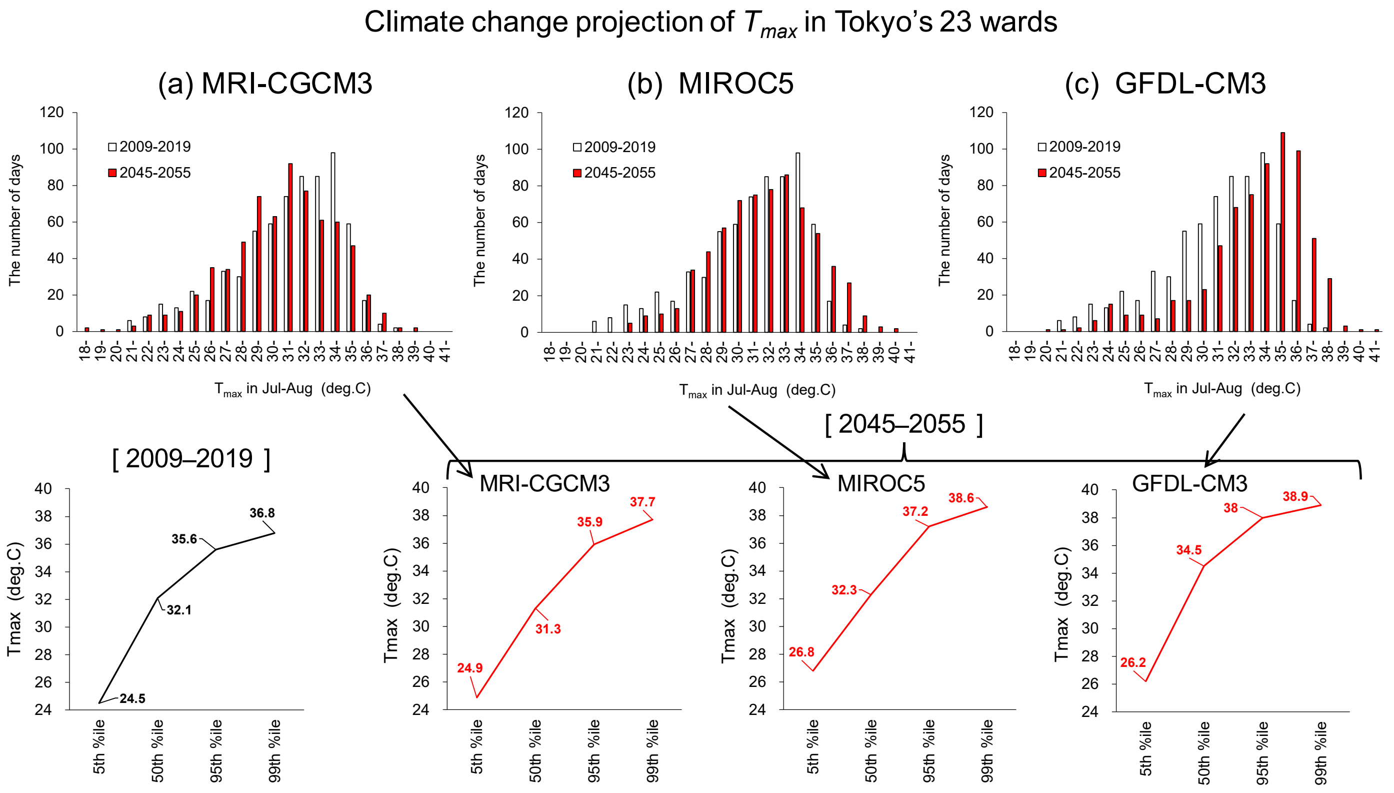

Supplementary Figure S12. Frequency distributions (upper panels) and percentiles (lower panels) of T_{max} in Tokyo's 23 wards. The results in 2009–2019 and climate projections (2045–2055) of (a) MRI-CGCM3, (b) MIROC5, and (c) GFDL-CM3 under the condition RCP8.5.
